# Dichotomy in hypoxia-induced mitochondrial fission in placental mesenchymal cells during development and preeclampsia: consequences for trophoblast mitochondrial homeostasis

**DOI:** 10.1038/s41419-022-04641-y

**Published:** 2022-02-26

**Authors:** Taylor Gillmore, Abby Farrell, Sruthi Alahari, Julien Sallais, Merve Kurt, Chanho Park, Jonathan Ausman, Michael Litvack, Martin Post, Isabella Caniggia

**Affiliations:** 1 Lunenfeld-Tanenbaum Research Institute, Sinai Health System, Toronto, ON M5T 3H7 Canada; 2 Institute of Medical Science, University of Toronto, Toronto, ON M5S 1A8 Canada; 3 Department of Physiology, University of Toronto, Toronto, ON M5S 1A8 Canada; 4 Translational Medicine Program, Peter Gilgan Centre for Research and Learning, The Hospital for Sick Children, Toronto, ON M5G1X8 Canada; 5 Department of Obstetrics and Gynecology, University of Toronto, Toronto, ON M5G 1E2 Canada

**Keywords:** Mechanisms of disease, Endocrine reproductive disorders

## Abstract

Dynamic changes in physiologic oxygen are required for proper placenta development; yet, when low-oxygen levels persist, placental development is halted, culminating in preeclampsia (PE), a serious complication of pregnancy. Considering mitochondria’s function is intimately linked to oxygen changes, we investigated the impact of oxygen on mitochondrial dynamics in placental mesenchymal stromal cells (pMSCs) that are vital for proper placental development. Transmission electron microscopy, proximity ligation assays for mitochondrial VDAC1 and endoplasmic reticulum IP3R, and immunoanalyses of p-DRP1 and OPA1, demonstrate that low-oxygen conditions in early 1st trimester and PE promote mitochondrial fission in pMSCs. Increased mitochondrial fission of mesenchymal cells was confirmed in whole PE placental tissue sections. Inhibition of DRP1 oligomerization with MDiVi-1 shows that low oxygen-induced mitochondrial fission is a direct consequence of DRP1 activation, likely *via* HIF1. Mitophagy, a downstream event prompted by mitochondrial fission, is a prominent outcome in PE, but not 1st trimester pMSCs. We also investigated whether mesenchymal–epithelial interactions affect mitochondrial dynamics of trophoblasts in PE placentae. Exposure of trophoblastic JEG3 cells to exosomes of preeclamptic pMSCs caused heightened mitochondrial fission in the cells *via* a sphingomyelin-dependent mechanism that was restored by MDiVi-1. Our data uncovered dichotomous regulation of mitochondrial fission and health in human placental mesenchymal cells under physiologic and pathologic hypoxic conditions and its impact on neighboring trophoblast cells.

## Introduction

Mitochondria are critical organelles responsible for cell survival and death [1, 2]. Importantly, not all mitochondria “are created equal”, and changes in their morphology is a central determinant of their capacity to generate essential ATP or to produce harmful byproducts, such as reactive oxygen species (ROS) [3]. Specifically, through cycles of mitochondrial fusion and fission—collectively termed mitochondrial dynamics—mitochondria respond to the requirements of the cell, and adjust their metabolism accordingly [4]. Classically, mitochondrial fusion enables mitochondrial DNA sharing and generates healthy mitochondria with enhanced energy production capabilities, whereas mitochondrial fission segregates fragmented and damaged mitochondria with impaired membrane potentials [1]. During fusion, outer mitochondrial membrane (OMM) proteins, mitofusins 1/2 (MFN1/MFN2), promote OMM merging of two adjacent mitochondria [5] that is followed by inner mitochondrial membrane fusion mediated by optic atrophy 1 (OPA1) protein. Conversely, mitochondrial fission is orchestrated by dynamin-related protein 1 (DRP1), a cytosolic protein that is recruited to the OMM to induce mitochondrial division through a GTP-dependent conformational change [6]. DRP1 functional activity is regulated by several posttranslational modifications; most notably, phosphorylation at serine 616 residue [7]. DRP1 recruitment to OMM is not random; in fact, ER tethering to mitochondria involves specific regions termed mitochondria-associated membranes (MAM) that contribute to the initiation of mitochondrial fission [8].

Alterations in mitochondrial dynamics underlie several diseases [9]. We have reported increased mitochondrial fission in placentae from pregnancies complicated by preeclampsia (PE) [10], a serious hypertensive disorder that affects 5–8% of all pregnancies and is a leading cause of maternal and fetal mortality and morbidity worldwide [11]. In particular, we showed heightened mitochondrial fission events in trophoblast cells of PE placentae, and attributed this to elevated mitochondrial ceramide and its induction of pro-apoptotic BOK [10]. Placental mesenchymal stromal cells (pMSCs) have been less studied but are vital for shaping a healthy placenta [12–15]. Therefore, we sought to examine the impact of oxygen on mitochondrial dynamic events in pMSCs across gestation. As placental hypoxia typifies PE [16, 17], we also investigated mitochondrial dynamic events in preeclamptic pMSCs. Studies have highlighted the role of trophoblast derived exosomes in pregnancy [18], yet the contribution of PE pMSC-secreted exosomes remains elusive. Hence, we also examined the effect of exosomes released by PE pMSCs on mitochondrial dynamics of neighboring trophoblast cells.

## Materials/subjects and methods

### Human placenta tissue collection

Informed consent was obtained from each individual. Tissue collection was carried out in agreement with ethics guidelines (University of Toronto’s Faculty of Medicine and Mount Sinai Hospital) by the Research Center for Women’s and Infants’ Health Biobank. First trimester placentae (6–12 weeks, *n* = 8) were collected at once from elective termination of pregnancies. PE subjects (*n* = 7) were selected on the basis of American College of Obstetrics and Gynecology clinical and pathological criteria [19]. Patients presenting with chronic hypertension, diabetes, infection, and kidney disease were excluded from the study. Normotensive patients with no signs of disease or pregnancy complications who delivered preterm (*n* = 4) and at term (*n* = 6) were used as controls. Control and pathological placentae were obtained from singleton pregnancies without fetal anomalies or chromosomal abnormalities. Preterm deliveries were primarily due to idiopathic labor, cervical incompetence, and preterm premature rupture of membranes. Clinical parameters of PE and normotensive control subjects are listed in Supplementary Table 1.

### Placental mesenchymal cells isolation and culture

Placental mesenchymal cells were isolated from first trimester, term, and PE placentae (for details on isolation procedures and treatments see Supplementary Methods). Isolated pMSCs were cultured at an oxygen tension that corresponded to their physiological oxygen environment at time of isolation. Specifically, pMSCs isolated from 6 to 8-week placenta and PE placenta were cultured at 3% O_2_, while pMSCs isolated from 10 to 12-week and term placenta were cultured at 8% O_2_. Exosomes were isolated from 24 h conditioned media of term and PE pMSCs as previously reported [20]. For details on isolation, characterization and uptake of exosomes see Supplementary Methods.

### Fluorescent activated cell sorting

Characterization of isolated pMSCs from 1st trimester, term and PE placentae was performed using FACS analysis as reported [20] (details in Supplementary Methods).

### JEG3 cell treatments

Human Choriocarcinoma JEG3 cells (ATCC, Manassas, VA, USA) JEG cells were used as a trophoblast model to study mitochondrial homeostasis and cell death [10, 21, 22]. Cells were grown under standard conditions (ambient air, 5% CO_2_) in Eagle’s Minimum Essential Medium (EMEM) supplemented with 10% (v/v) FBS and 1% (v/v) P/S. JEG3 cells were treated with 25 μM Imipramine (Sigma-Aldrich, St. Louis, USA #I7379) or 10 μM Fluoxetine (Sigma-Aldrich, St. Louis, USA-# F132) for 6–12 h prior to fixation for IF or PLA measurements as previously described [20]. JEG3 cells seeded onto 6-well plates were exposed to 5–20 μg/mL sphingomyelin 18:0 (SM-18:0 (d18:1/18:0) Sigma-Aldrich, USA, #860586 P) or 90% EtOH vehicle for 6 h prior to immunofluorescence (IF) and western blot (WB) analyses. Exosomes (2.5 × 10^7^) from PE or term pMSCs were added to JEG3 cells and incubated for 3, 6, and 24 h. Cells were then either harvested for mitochondrial isolations or fixed with 3.7% (v/v) paraformaldehyde for IF analysis.

### MitoSOX and MitoTracker CMXRos

Five μM of MitoSOX^TM^ Red Mitochondrial Superoxide Indicator (M36008, Thermo Fisher Scientific, Waltham, MA, USA) was used to examine ROS production in term, PE and 1st trimester pMSCs cultured at 3 and 8% O_2_. MitoTracker^TM^ Red CMXRos (M7512, Thermo Fisher Scientific, Waltham, MA, USA) was used to visualize mitochondria in live cells as previously reported [10]. Cells treated with MitoSOX or MitoTracker were washed with PBS before being mounted and imaged using a Leica SD6000 spinning disk confocal microscope (Leica Camera, Wetzlar, Germany).

### Transmission electron microscopy (TEM)

Placental tissue from PE and control pregnancies and pMSCs cells were processed for TEM as reported [10, 21] (details in Supplementary Methods).

### Mitochondria isolation

Mitochondria were isolated as previously described [10]. Purity was validated by WB for TOM20 and VDAC1 [10].

### Western blot and immunofluorescence analyses

Following isolation and treatments, 1st trimester, term, and PE pMSCs were lysed in RIPA buffer or processed for IF staining. WB and IF were performed as previously described [10]. ACTB, TOM20, VDAC1, CD63 were used as loading controls. Densitometry was carried out using Image J software (NIH). IF images were analyzed using Volocity Imaging software and Image J software. Original uncropped WB are reported in Supplementary Material.

Following respective treatments, pMSCs were fixed and processed as previously described [21]. Cells were incubated overnight with primary antibodies at 4 °C. Cells were then incubated for 1 h with Alexa Flour^®^-conjugated secondary antibodies. Cells were imaged using confocal microscopy. For details on antibodies used for IF and WB see Supplementary Materials.

### Proximity ligation assay

First trimester, PE, and Term pMSCs as well as JEG3 cells cultured under the appropriate conditions were fixed in ice-cold 3.7% (v/v) paraformaldehyde and permeabilized with 0.2% (v/v) Triton X-100. The Duolink^®^ PLA Fluorescence Protocol (Sigma-Aldrich) was used as described [23] (see details in Supplementary Methods).

### SMPD1 activity assay

SMPD1 activity in JEG3 cells was measured as reported [24] (see details in Supplementary Methods).

### Lipid mass spectral analysis

Ceramide and sphingomyelin species in pMSC-derived exosomes and mitochondria were quantified using high performance liquid chromatography coupled to tandem mass spectrometry at the Analytical Facility for Bioactive Molecules at the Hospital for Sick Children, Toronto as described [10, 24, 25].

### Statistical analysis

Statistical analysis was done using GraphPad Prism software. Nonparametric Mann–Whitney test was used to compare two groups with *n* > 3. Two-tailed unpaired Student’s *t* test was used to compare two groups with *n* = 3. All data presented are represented as the mean ± SEM. Statistical significance was indicated as *p < 0.05; **p < 0.01; ***p < 0.001.

## Results

### Mitochondrial fission is increased in pMSCs isolated from early 1st trimester placenta

**Fig. 1:**
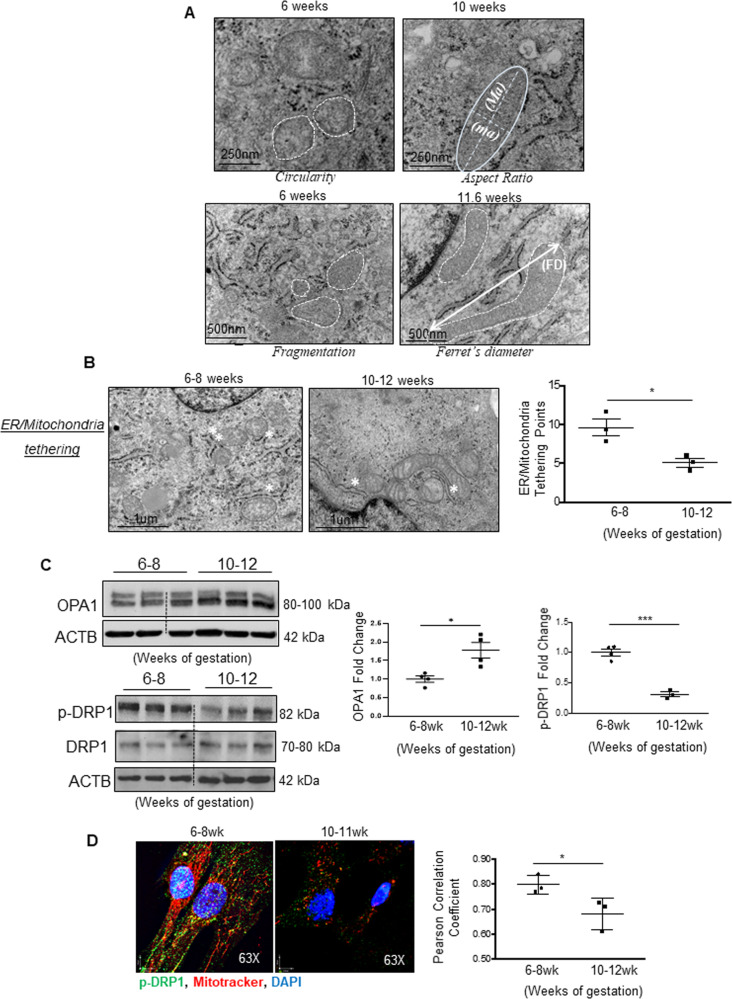
Mitochondrial dynamics in first trimester placental mesenchymal stromal cells. **A** Representative transmission electron (TEM) images of pMSCs isolated from placentae of 6–8 (*n* = 4 separate isolations) and 10–12 (*n* = 3 separate isolations) weeks of gestation. Mitochondria are outlined by dotted lines and their measurements are indicated. Scale bar depicts size. **B** Representative TEM images of pMSCs from 1st trimester placentae showing endoplasmic reticulum (ER) to mitochondria tethering points (white asterisks). Scale bar indicates size. Right graph shows average ER/mitochondria tethering points per image (4 images per pMSC isolation) of pMSCs from placentae of 6–8 (*n* = 3 separate isolations) and 10–12 (*n* = 3 separate isolations) weeks of gestation, *p < 0.05, unpaired student’s *t* test. **C** Representative Western blots for OPA1 and p-DRP1 in lysates of pMSCs isolated from placentae of 6–8 (*n* = 4) and 10–12 (*n* = 3) weeks of gestation. Densitometry data are normalized to ACTB and expressed as a fold change relative to pMSCs isolated from 6 to 8-week placentae, *p < 0.05, unpaired student’s *t* test. **D** Representative immunofluorescence confocal images and quantification of co-localization by Pearson correlation coefficient of p-DRP1 (green) and Mitotracker (red) in pMSCs isolated from placentae of 6–8 (*n* = 3) and 10–12 (*n* = 3) weeks of gestation, *p < 0.05, unpaired student’s *t* test. Nuclei are visualized with DAPI (blue).

We first examined mitochondrial dynamic events in pMSCs isolated from placentae across the 1st trimester of gestation. Consistent with criteria that identify pMSCs [26], isolated cells stained positive for CD29, CD73, CD90 and CD105, and negative for CD34 and CD45 (Supplementary Fig. 1A). TEM was employed for assessment of mitochondrial morphology [21]. Heightened mitochondrial fission events (i.e., presence of small circular mitochondrial fragments) were observed in pMSCs from placentae of 6–8 weeks of gestation compared to pMSCs from 10 to 12 weeks (Fig. 1A). These morphologic differences prompted us to quantitatively assess mitochondria morphology by established parameters [21]. Specifically, mitochondria were assessed for *size* [perimeter and surface area], *shape* [aspect ratio (“length-to-width” ratio), *circularity* (value of 1 = perfect circle)], and *fragmentation* [*Feret’s diameter* (longest distance between any two points in the mitochondria)] as well as total number of mitochondria per TEM image. Compared to mitochondria from 10 to 12-week pMSCs, mitochondria from 6 to 8-week pMSCs were smaller (decreased surface area and perimeter); more globular (smaller aspect ratio and circularity values approaching those of a perfect circle); and more fragmented (decreased Feret’s diameter and increased number of mitochondria per image) (Fig. 1A and Supplementary Table 2). Consistent with this morphologic evidence of heightened fission, 6–8 weeks pMSCs exhibited more endoplasmic reticulum (ER)-to-mitochondrial tethering points than 10–12 weeks pMSCs (Fig. 1B). WB for fusion (OPA1) and fission (p-DRP1; phosphorylated at serine 616) and total DRP1) markers corroborated elevated mitochondrial fission in 6–8 weeks compared to 10–12 weeks pMSCs. Specifically, OPA1 levels were decreased while p-DRP1 was increased (Fig. 1C). IF analysis confirmed increased expression and localization of p-DRP1 to mitochondria (labeled by MitoTracker^TM^ red) in 6–8 relative to 10–12 weeks pMSCs (Fig. 1D).

### Low-oxygen promotes mitochondrial fission

**Fig. 2:**
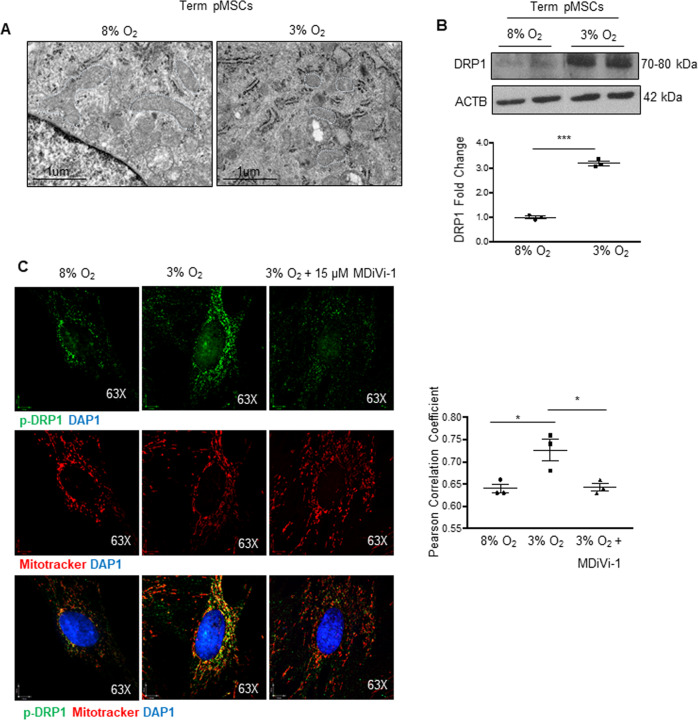
Low-oxygen promotes mitochondrial fission in term placental mesenchymal stromal cells. **A** Representative TEM images of mitochondria of pMSCs isolated from term placentae and cultured at either 8% (*n* = 6 separate isolations) or 3% O_2_ (*n* = 3 separate isolations). Mitochondria are outlined by dotted lines. Scale bar indicates size. **B** Representative western blot for DRP1 and associated densitometry in term pMSCs cultured at 8% (*n* = 3) and 3% O_2_ (*n* = 3) for 24 h. Densitometry data are normalized to ACTB and expressed as fold change relative to 8% O_2,_ ***p < 0.001, unpaired student’s *t* test. **C** Representative immunofluorescence confocal images and quantification of co-localization by Pearson correlation coefficient of p-DRP1 (green) and Mitotracker (red) in term pMSCs cultured at either 8% O_2_ (*n* = 3), 3% O_2_ (*n* = 3), or 3_%_ O_2_ with 15 μM MDiVi-1 (*n* = 3) for 24 h, *p < 0.05, unpaired student’s *t* test. Nuclei are visualized with DAPI (blue).

We next investigated the involvement of oxygen in regulating mitochondrial dynamics in pMSCs. TEM showed striking mitochondrial morphologic differences between term pMSCs cultured at 3% O_2_ *versus* physiologic 8% O_2_ (Fig. 2A). Mitochondria appeared more fragmented in term pMSCs when cultured at 3% O_2_ and exhibited decreased surface area, perimeter, and Feret’s diameter (Fig. 2A and Supplementary Fig. 1C). Greater DRP1 and mitochondrial p-DRP1 levels corroborated the increased fission at 3% O_2_ (Fig. 2B, C). To determine if low oxygen-induced fission is due to recruitment and phosphorylation of DRP1 to mitochondria we employed the mitochondrial division inhibitor 1 (MDiVi-1) that blocks DRP1 oligomerization and GTPase activity at the mitochondria [27]. Treatment of term pMSCs with 15 μM MDiVi-1 (optimal non-toxic dosage of MDiVi-1 established in pilot experiments (Supplementary Fig. 2A, B) indeed inhibited the increase in mitochondrial p-DRP1 as measured by IF in cells exposed to 3% O_2_ (Fig. 2C).

### Mitochondrial morphology is altered in pMSCs from PE placentae

**Fig. 3:**
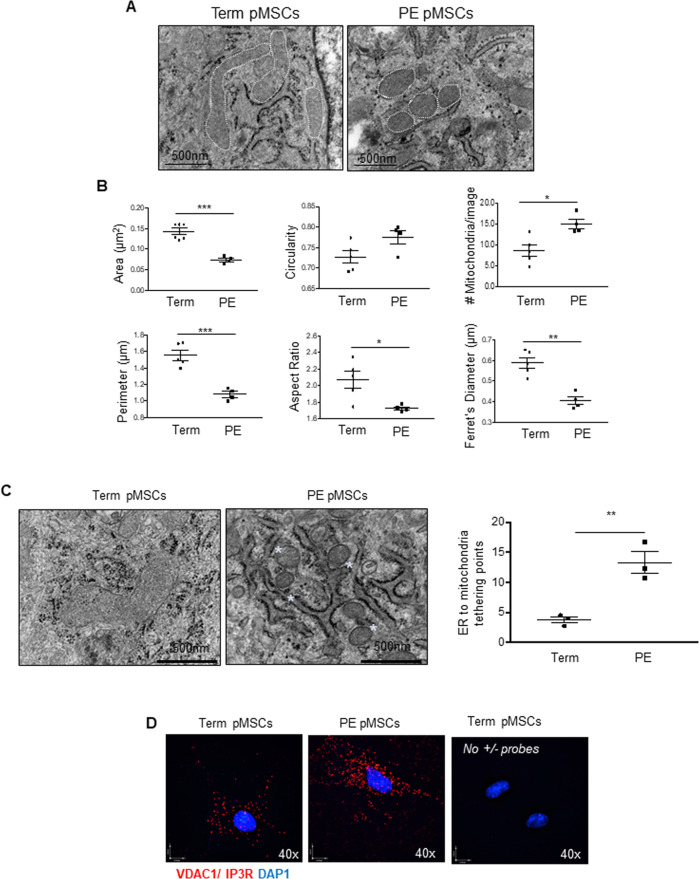
Mitochondria dynamics and ER-to-mitochondrial tethering in term and preeclamptic (PE) placental mesenchymal stromal cells. **A** Representative TEM images of pMSCs isolated from term (*n* = 5 separate isolations) and PE (*n* = 4 separate isolations) placentae. Mitochondria are identified by white dotted lines. Scale bar indicates size. **B** Mitochondria parameters (surface area, perimeter, Ferret’s diameter, aspect ratio, number of mitochondria, and circularity) in pMSC isolated from PE (*n* = 4) and Term (*n* = 6) placentae, *p < 0.05; **p < 0.01; ***p < 0.001, unpaired student’s *t* test. **C** Representative TEM images and quantification of ER-to mitochondria tethering in pMSCs isolated from term (*n* = 3) and PE (*n* = 3) placentae. Proximity of the ER-to mitochondria is indicated by white asterisks. Scale bar indicates size. **D** Representative immunofluorescence images of in situ proximity ligation assay targeting VDAC1 and IP3R interactions in pMSCs isolated from term and PE placentae. Each individual interaction is represented by a red dot. Nuclei are stained with DAPI (blue). Reactions without positive and negative probes in term and PE pMSCs are negative controls. PLA assay was repeated twice with similar results.

**Fig. 4:**
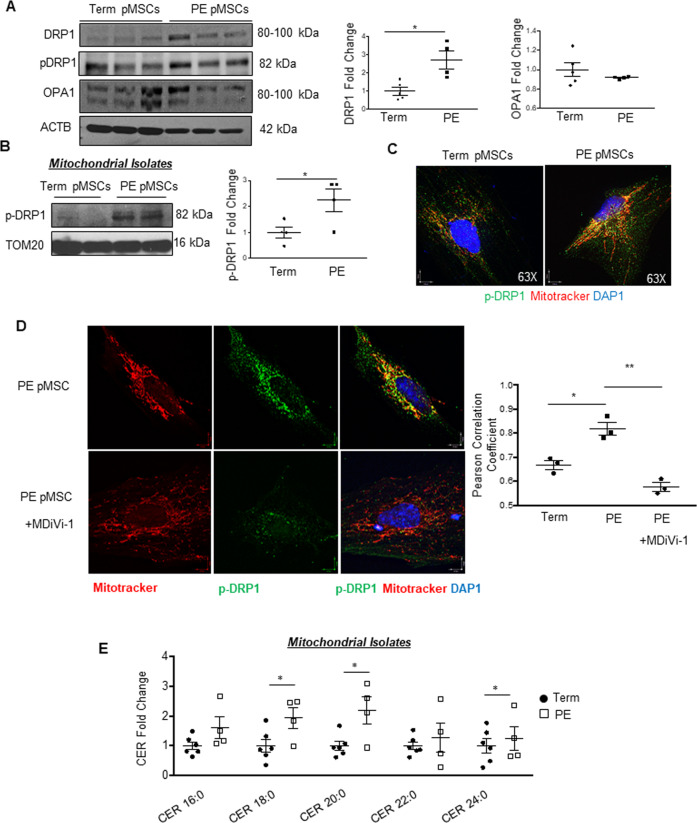
Placental mesenchymal stromal cells isolated from preeclamptic (PE) placentae exhibit increased mitochondrial fission. **A** Representative Western blots for DRP1, p-DRP1 and OPA1 and associated densitometry in pMSCs isolated from term (*n* = 5 separate isolations) and PE (*n* = 4 separate isolations) placentae. Densitometry data are normalized to ACTB and expressed as fold change relative to term pMSCs, *p < 0.05, unpaired student’s *t* test. **B** Representative Western blots for p-DRP1 and TOM20 and associated densitometry in mitochondrial isolates (MI) from pMSCs of term (*n* = 4) and PE (*n* = 4) placentae. Densitometry data are normalized to TOM20 and expressed as fold change relative to term. **C** Representative immunofluorescence confocal images of p-DRP1 (green) and Mitotracker (red) in pMSCs isolated from term and PE placentae. Nuclei are visualized with DAPI (blue). **D** Representative immunofluorescence confocal images and quantification of co-localization by Pearson correlation coefficient of p-DRP1 (green) and Mitotracker (red) in term and PE pMSCs, and PE pMSC treated with 15 μM MDiVi-1, *p < 0.05; **p < 0.01, unpaired student’s *t* test, *n* = 3 separate pMSC isolations. Nuclei are stained with DAPI. **E** Targeted sphingolipid analysis of ceramide species in isolated mitochondria of pMSCs from PE (*n* = 4) and term (*n* = 6) placentae. Values are presented as fold changes relative to term pMSCs, *p < 0.05, unpaired student’s *t* test.

Impaired oxygen sensing leading to placental hypoxia characterizes PE [16, 17]. Therefore, we isolated pMSCs from PE and term placentae and cultured them in their standard physiologic oxygen conditions (term: 8% O_2_ and PE: 3% O_2_). Isolated pMSCs from PE placentae displayed similar cell surface marker characteristics as pMSCs from 1st trimester and term placentae (Supplementary Fig. 1B vs. 1A). TEM revealed that compared to term pMSCs, mitochondria from PE pMSCs were markedly smaller (Fig. 3A). Quantitatively, mitochondria of PE pMSCs had a reduced surface area and perimeter and were more globular, as indicated by aspect ratio and circularity values closer to that of a perfect circle (Fig. 3B). In addition, mitochondria of PE pMSCs were more fragmented as demonstrated by decreased Feret’s diameter and increased total number of individual mitochondria per image (Fig. 3B). TEM of whole placental tissue sections verified heightened mitochondrial fission events in mesenchymal cells of PE vs. preterm age-matched control (PTC) placentae in situ (Supplementary Fig. 3A). ER-to-mitochondrial tethering, a necessary pre-requisite for the initiation of fission [8], was found to be significantly increased in pMSCs isolated from PE placentae compared to term pMSCs (Fig. 3C). To conclusively prove this interaction, we performed a proximity ligation assay (PLA) targeting the ER-associated inositol triphosphate receptor (IP3R) and the mitochondrial voltage dependent anion channel 1 (VDAC1), two proteins known to form contacts at the MAM interface [28]. PLA showed a marked increase in the number of VDAC1/IP3R interaction points (red signals) in pMSCs from PE compared to term (Fig. 3D). In support of augmented fission, DRP1 (and p-DRP1) levels were significantly higher in PE than term pMSC lysates, while OPA1 levels were, although not significantly, reduced (Fig. 4A). No changes in p-DRP1 or OPA1 were observed between PTC and term pMSC or whole lysates from PTC and term placentae (Supplementary Fig. 4A). Also, mitochondria isolated from PE pMSCs contained significantly more p-DRP1 than those from term pMSCs (Fig. 4B). Furthermore, IF showed marked co-localization of p-DRP1 with mitochondria in PE compared to term pMSCs (Fig. 4C). Treatment with MDiVi-1 revoked p-DRP1 recruitment to mitochondria in PE pMSCs (Fig. 4D). Previously we have reported that an accumulation of ceramides in mitochondria from PE placentae promotes mitochondrial fission [10]. Therefore, we hypothesized that mitochondria of pMSCs from PE placentae should have elevated ceramide levels. Indeed, lipidomic mass spectrometry revealed significant increases in Cer 18:0, Cer 20:0, and Cer 24:0 species in mitochondria of pMSCs isolated from PE *versus* term placentae (Fig. 4E).

### Reactive oxygen species and mitophagy are increased in PE pMSCs

**Fig. 5:**
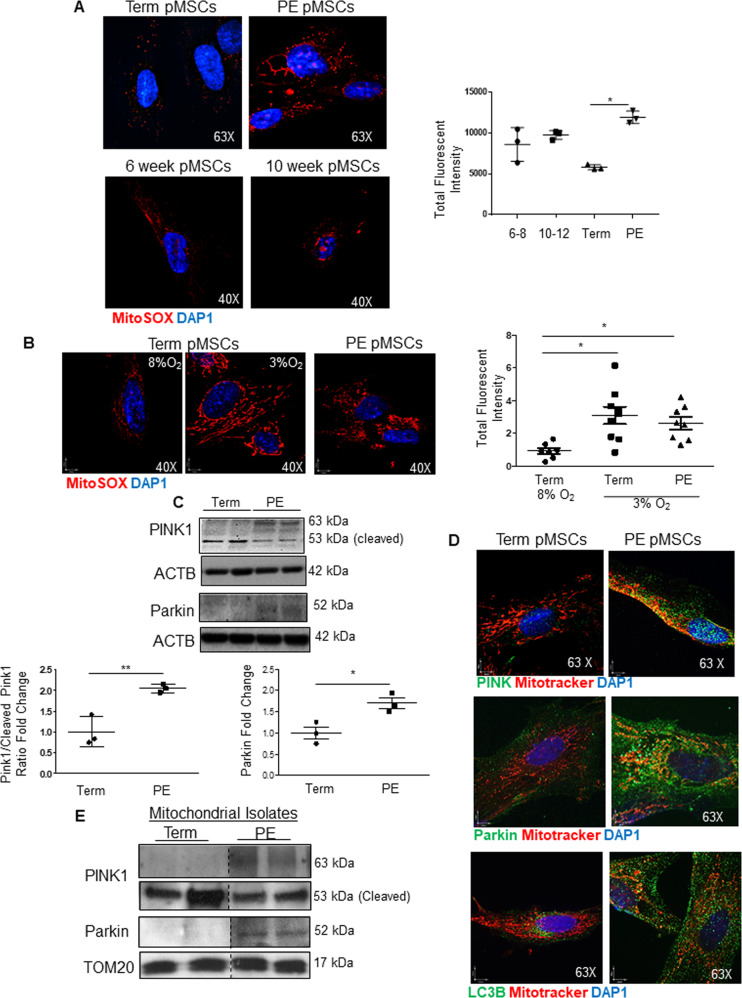
Enhanced mitochondrial ROS production in preeclamptic (PE) placental mesenchymal stromal cells is associated with increased mitophagy. **A** Representative immunofluorescence confocal images and quantification of mitochondrial ROS (red) production using mitoSOX in pMSCs isolated from 6 to 8-week (*n* = 3), 10 to 12-week (*n* = 3), term (*n* = 3), and PE (*n* = 3) placentae. Nuclei are visualized with DAPI (blue). *p < 0.05, unpaired student’s *t* test. **B** Representative immunofluorescence confocal images for mitochondrial ROS (red) production using mitoSOX in term pMSCs cultured at 8% and 3% O_2_ in comparison to PE pMSCs maintained at 3% O_2_. Experiment repeated three times with similar results. **C** Representative Western blots for PINK1 and Parkin and associated densitometry in pMSCs isolated from 6 to 8-week (*n* = 3), term (*n* = 3), and PE (*n* = 3) placentae. PINK1 is quantified as a ratio of uncleaved (63 kDa) active PINK1 to cleaved (53 kDa) inactive PINK1 and expressed as fold change relative to term pMSCs. Both PINK1 and Parkin are normalized to ACTB. *p < 0.05; **p < 0.01, unpaired student’s *t* test. **D** Representative immunofluorescence confocal images of PINK (green) and mitotracker (red); Parkin (green) and mitotracker (red) and LC3B and mitotracker (red) in term, and PE pMSCs. Nuclei are stained with DAPI (blue). **E** Representative Western blots for PINK1 and Parkin in mitochondrial isolates from term and PE pMSCs; TOM20 was used as control.

**Fig. 6:**
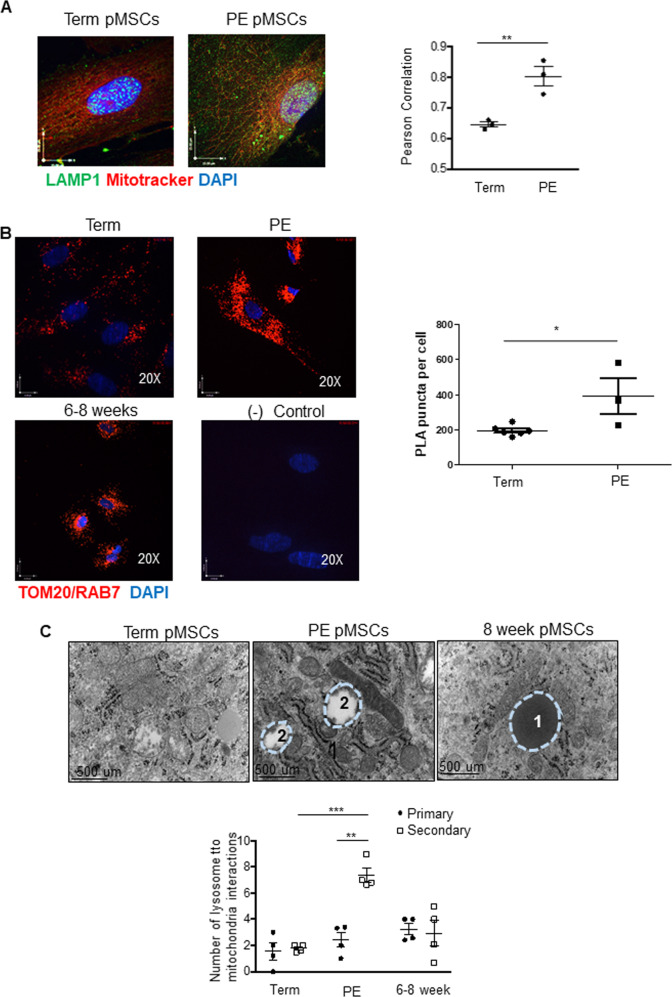
Augmented lysosome-to-mitochondria interactions in preeclamptic (PE) placental mesenchymal stromal cells. **A** Representative immunofluorescence confocal images and quantification of co-localization by Pearson correlation coefficient of lysosome marker LAMP1 (green) and mitotracker (red) in pMSCs isolated from term (*n* = 3) and PE (*n* = 3) placentae,*p < 0.05; **p < 0.01, unpaired student’s *t* test. Nuclei are stained with DAPI (blue). **B** Proximity ligation assay of TOM20 and RAB7 and its quantification in, term, PE and 6–8-week pMSCs depicting in situ lysosome and mitochondria interactions (red). Reactions without positive and negative probes in term pMSCs are negative controls. Nuclei are stained with DAPI (blue). **C** Representative TEM images and quantification of lysosome (1 = primary lysosome, 2 = secondary lysosome) to mitochondria interactions in 6–8-week (*n* = 4), term (*n* = 4), and PE (*n* = 4) pMSCs. **p < 0.01; ***p < 0.001, 2-way ANOVA with Bonferroni multiple comparisons. Scale bar indicates size.

Next, we assessed mitochondrial function by measuring mitochondrial ROS (mtROS) production through MitoSOX, a fluorogenic dye that exclusively targets mitochondria and measures their superoxide anion generation [29]. IF showed significantly greater mtROS generation in PE pMSCs compared to term pMSCs (Fig. 5A). In contrast, no significant change in mtROS generation was observed between 6–8-week and 10–12-week pMSCs. When term pMSCs were maintained at 3% O_2_ (hypoxic conditions for term pMSCs), they increased their mtROS production to levels found in PE pMSCs (Fig. 5B). High levels of ROS production can exceed the mitochondria’s antioxidant capacity, leading to their damage. In this scenario, mitochondria employ mitophagy, a specialized form of autophagy, to remove these damaged organelles. Fission functions upstream of mitophagy by sequestering damaged mitochondria that are then targeted by the PINK/Parkin complex for subsequent disposal. When a mitochondrion loses its membrane potential, PINK1 fails to be internalized into the mitochondrial matrix where it gets cleaved, and remains active on the OMM surface where it recruits Parkin [30]. The ratio of full-length, active PINK1 (63 kDa) to cleaved, inactive PINK1 (53 kDa) was increased in PE compared to term pMSCs (Fig. 5C) and this was accompanied by an increase in Parkin (Fig. 5C). Both PINK1 and Parkin co-localized to mitochondria in PE pMSCs; in contrast, Parkin did not localize to mitochondria in term pMSCs (Fig. 5D). Quantification of PINK1 co-localization with mitochondria revealed a significant increase in PE pMSCs (Pearson’s Correlation Coefficient of 0.3590 in term pMSCs vs. 0.5060 in PE pMSCs; Supplementary Fig. 4B).These data support active PINK1/Parkin-mediated mitophagy in PE pMSCs. Increased LC3B association to mitochondria in PE pMSCs corroborated the heightened mitophagy in these cells (Fig. 5D). In addition, WB showed that both PINK1 and Parkin protein levels in mitochondrial isolates were elevated in PE pMSCs (Fig. 5E), and this was accompanied by increased co-localization of Parkin with the downstream autophagy markers, LC3B and LAMP1 (Supplementary Fig. 4C). To further establish the occurrence of mitophagy in PE pMSCs, we investigated the final stages of this mode of cell death by examining lysosome-to-mitochondria interactions. IF revealed that the association of lysosomal-associated membrane protein 1 (LAMP1) with mitochondria was increased in PE *versus* term pMSCs (Fig. 6A). PLA of lysosome marker RAB7 and mitochondria marker TOM20 verified a greater interaction between these organelles in PE than term pMSCs (Fig. 6B). Interestingly, RAB7/TOM20 interactions were also detected in 6–8 weeks pMSCs, but not to the degree observed in PE pMSCs (Fig. 6B). TEM was employed to visualize and quantify primary and secondary lysosomes as previously described [20] as well as lysosome-mitochondria interactions in isolated pMSCs. While primary lysosome-to-mitochondria interactions were found in pMSCs from early 1^st^ trimester placentae, numerous secondary lysosome-to-mitochondria interactions (Fig. 6C) that are indicative of mitophagy [31] were observed in PE pMSCs.

### Exosomes derived from preeclamptic pMSCs affect mitochondrial dynamics in JEG3 cells

**Fig. 7:**
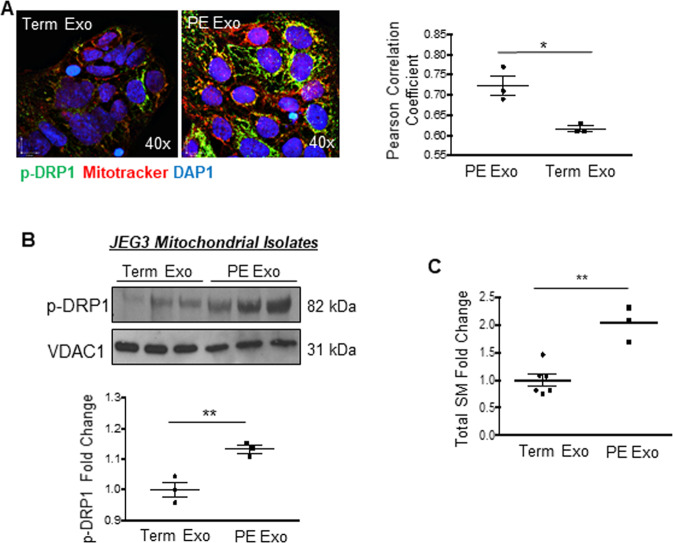
Exposure of trophoblastic JEG3 cells to exosomes from preeclamptic (PE) placental mesenchymal stromal cells tilt their mitochondrial dynamics towards fission. **A** Representative immunofluorescence confocal images and quantification of co-localization by Pearson correlation coefficient of p-DRP1 (green) and mitotracker (red) in JEG3 cells treated with exosomes secreted by PE and term pMSCs (*n* = 3 separate exosome and pMSC isolations). *p < 0.05, unpaired student’s *t* test. Nuclei are stained with DAPI (blue). **B** Representative western blot for p-DRP1 and VDAC1 in JEG3 cells treated with exosomes isolated from term and PE pMSCs. Densitometry quantification of p-DRP1 is normalized to VDAC1 and expressed as fold change relative to term EXO group. *p < 0.05, unpaired student *t* test, *n* = 3. **C** Targeted sphingolipidomic analysis of total sphingomyelin species in exosomes isolated from term (*n* = 6) and PE (*n* = 3) pMSC (*p < 0.05, unpaired Student’s *T* test).

**Fig. 8:**
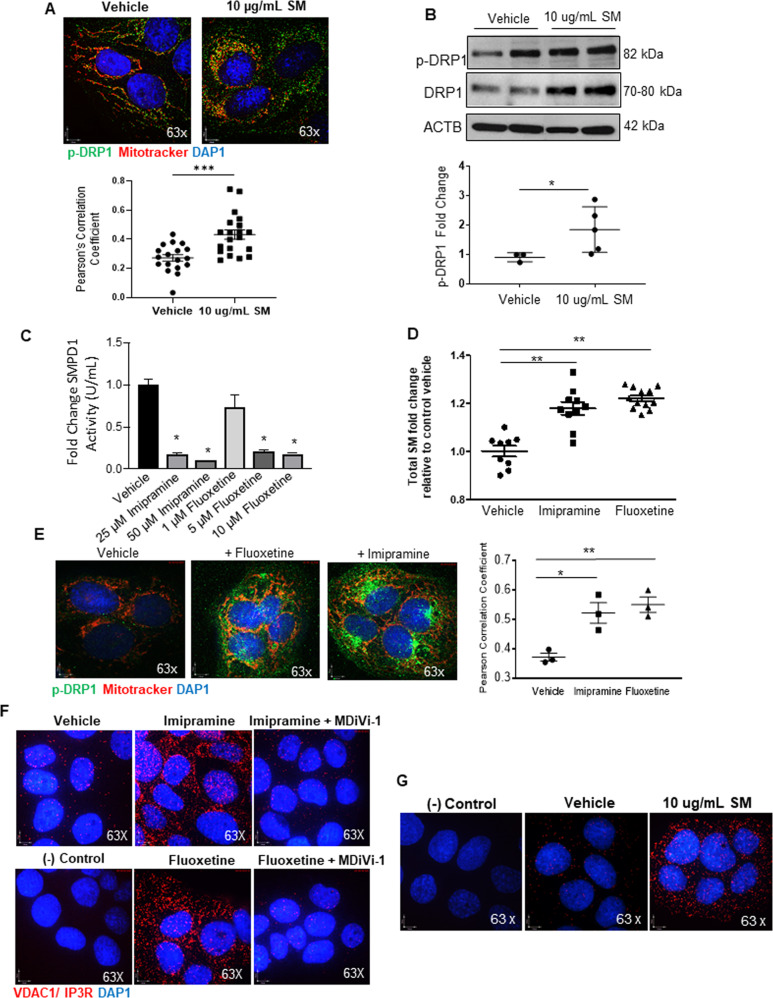
Exposure of trophoblastic JEG3 cells to sphingomyelin promotes mitochondrial fission. **A** Representative immunofluorescence confocal images and associated Pearson’s Correlation Coefficient analysis of p-DRP1 (green) and mitotracker (red) and in JEG3 cells treated with 10 µg/mL SM 18:0 for 6 h. Nuclei were stained with DAPI (blue). **B** Representative western blot for p-DRP1 and DRP1 in JEG3 cells treated with 10 µg/mL SM 18:0. Densitometry quantification for p-DRP1 is normalized to ACTB and expressed as fold change relative to vehicle controls. *p < 0.05, unpaired Student *T* test, *n* = 3. **C** SMPD1 activity assay in JEG3 cells following imipramine and fluoxetine exposure. **D** Targeted LC-MS/MS analysis of sphingomyelin species in JEG3 cells exposed to acid sphingomyelinase inhibitors, imipramine (25 μM) and fluoxetine (5 μM) for 6 h. **E** Representative IF analysis and quantification of co-localization by Pearson correlation coefficient of p-DRP1 (green) and mitotracker (red) in JEG3 cells treated with acid sphingomyelinase inhibitors imipramine (25 μM) and fluoxetine (5 μM) for 6 h. Nuclei were stained with DAPI (blue). **F** Representative immunofluorescence images of in situ proximity ligation assay targeting VDAC1 and IP3R interactions in JEG3 cells cultured in the presence and absence of imipramine and fluoxetine. Each individual interaction is represented by a red dot. Nuclei were stained with DAPI (blue). Reactions without negative probe in cells exposed to vehicle were used as negative controls. **G** Representative immunofluorescence images of proximity ligation assay targeting VDAC1 and IP3R association in JEG3 cells treated with 10 μg/mL sphingomyelin for 6 h.

Emerging evidence indicates that villous mesenchymal cells communicate *via* exosomes with neighboring placental cells [32]. Hence, we questioned whether exosomes secreted by pMSCs could impair mitochondrial dynamic events in trophoblast cells. Exosomes isolated from media conditioned by term and PE pMSCs were characterized by Nanosight tracking analysis (NTA) and expression of cell surface markers CD9 and CD63 (Supplementary Fig. 5A, B). Fluorescence analysis revealed a rapid uptake (≥30 min) of the pMSC-derived exosomes by trophoblastic JEG3 cells (Supplementary Fig. 5C). Exposure of JEG3 cells to exosomes released by PE pMSCs (2.5 × 10^7^ particles/2 × 10^5^ cells per well) increased mitochondrial p-DRP1 expression and localization compared to cells exposed to exosomes derived from term pMSCs (Fig. 7A and Supplementary Fig. 5D). In pilot experiments, no discernable changes were observed in p-DRP1 between vehicle treated and term exosome-treated JEG3 cells (Supplementary Fig. 5E). Phosphorylated DRP1 levels were increased in mitochondria isolated from JEG3 cells treated with exosomes from PE (PE Exo) *versus* term (Term Exo) pMSCs (Fig. 7B). We have recently reported that maternal circulating exosomes of trophoblastic origin in PE are enriched in sphingomyelin (SM) [25]. Therefore, we investigated the sphingolipid cargo and observed that exosomes from PE pMSCs contained twofold more SM than exosomes from term pMSCs (Fig. 7C). To understand whether sphingomyelin directly affects mitochondrial fission, we exposed JEG3 cells to 10 μg/mL synthetic SM 18:0. Treatment with SM 18:0 significantly enhanced p-DRP1 mitochondrial localization (Pearson’s Correlation Coefficient 0.2730 in vehicle vs. 0.4295 in SM treatment; Fig. 8A) and content (Fig. 8B) relative to vehicle-treated controls, implying a direct role for this lipid in influencing mitochondrial dynamics. Alternatively, internal sphingomyelin levels in JEG3 cells were raised by inhibiting acid sphingomyelinase (SMPD1), an enzyme that breaks down sphingomyelin to ceramide, with imipramine and fluoxetine [20, 33]. We first validated that both inhibitors blocked SMPD1 activity in JEG3 cells (Fig. 8C) and elevated cellular sphingomyelin levels (Fig. 8D). IF revealed that both SMPD1 inhibitors promoted p-DRP1 recruitment to mitochondria (Fig. 8E). Additionally, PLA showed increased tethering of ER-to mitochondria in JEG3 cells following SMPD1 inhibition as visualized by augmented VDAC1/IP3R interaction points that was reversed upon addition of MDiVi-1 (Fig. 8F). Direct exposure of JEG3 cells to sphingomyelin also resulted in an enhanced PLA signal for VDAC1/IP3R, indicating increased ER-mitochondria association (Fig. 8G). Treatment of JEG3 with sphingomyelin for 3 and 6 h did not affect cell viability and the expression of apoptotic markers p53 and BNIP3 (Supplementary Fig. 6).

## Discussion

In the present study, we demonstrate striking oxygen-dependent changes in mitochondrial morphology during early placental development and report that low-oxygen promotes mitochondrial fission events in healthy placental mesenchymal cells (pMSCs). In line with these findings, we found that mitochondrial fission events are dominant in pMSCs isolated from preeclamptic placentae. However, the role of physiologic and pathologic hypoxia in mitochondrial fission and health is dichotomous. Low-oxygen-mediated mitochondrial fission in early 1st trimester pMSCs leads to the production of small, functional mitochondria with limited ROS production while chronic hypoxia in PE augments mitochondrial fission in pMSCs due to dysfunctional ROS producing mitochondria that are disposed *via* mitophagy. The opposing mitochondrial fission outcomes between early 1st trimester and preeclamptic pMSCs are a consequence of mitochondria interacting with distinct lysosome sub-populations (primary *versus* secondary). Furthermore, we show that preeclamptic pMSCs *via* exosomes can alter the balance of mitochondrial dynamic events in neighboring trophoblast cells towards fission.

Placentae from early (<10 weeks) gestation and from preeclamptic pregnancies share similarities, including a low oxygenated environment [34]. At around 10–12 weeks of gestation, the maternal spiral arteries remodel into high caliber conduits capable of sustaining increased blood flow, and this associates with a physiological rise in oxygen tension [35]. Here, we demonstrate that this rise in oxygenation is accompanied by a change in mitochondrial morphology of pMSCs. Mitochondria of 6–8-week pMSCs are small and globular (indicative of fission) while mitochondria of 10–12-week pMSCs are more elongated and interconnected (indicative of fusion). In line with studies using pancreatic beta INS-1E cells and human pulmonary artery smooth muscle cells [36, 37], we observed augmented mitochondrial fission in term pMSCs after exposure to hypoxia. Our finding of increased mitochondrial fission in pMSCs isolated from hypoxic preeclamptic placentae agrees with reports showing excessive mitochondrial fragmentation and p-DRP1 expression in PASMCs from patients with pulmonary hypertension that suffer hypoxemia [38]. Thus, changes in mitochondrial morphology towards fission in pMSCs from early 1st trimester placenta and in PE are a consequence of a low-oxygen environment. Hypoxia inducible-transcription factor (HIF1), a key oxygen sensor and regulator of oxygen homeostasis in the human placenta, is highly expressed in early 1st trimester and persistently upregulated in PE [16, 17, 34]. HIF1 regulates the expression and posttranslational modification of mitochondrial proteins, including critical fission protein DRP1 [39] by regulating its phosphorylation at Serine 616 [40, 41]. We speculate that elevated HIF1 levels in early 1st trimester and preeclamptic placentae are responsible for DRP1 activation resulting in heightened mitochondrial fission. The finding of DRP1 inhibitor MDiVi-1 abrogating mitochondrial p-DRP1 recruitment in both term pMSCs kept at 3% O_2_ and preeclamptic pMSCs supports the idea that hypoxia-mediated upregulation of mitochondrial fission involves a DRP1-dependent mechanism.

Provided that hypoxia in development is a physiologic necessity, whereas in PE it is both a consequence and contributor to disease pathology, we reasoned that the downstream effects of higher fission rates, particularly the disposal of dysfunctional fragmented mitochondria *via* mitophagy, may differ. Signs of PINK1/Parkin-mediated mitophagy were greater in preeclamptic than term pMSCs. During the final steps of mitophagy, autophagosome fusion to a primary lysosome activates lysosomal enzymes leading to the generation of a secondary lysosome that permits mitochondria degradation. Hence, secondary lysosomes are associated with cell death events, whereas primary lysosomes have traditionally been viewed as passive organelles, housing inactive enzymes [20]. Our PLA for RAB7 and TOM20 demonstrated a strong association of lysosomes with mitochondria in preeclamptic pMSCs and to a lesser extent in 6–8-week pMSCs. Ultrastructural analysis by TEM revealed primary lysosome-to-mitochondria interactions in 6–8-week pMSCs, while prominent secondary lysosome-to-mitochondria interactions were noted in preeclamptic pMSCs. These findings are consistent with heightened mitochondrial fission in PE pMSCs triggering downstream mitophagy events. Primary lysosome-to-mitochondria interactions have been reported to initiate mitochondrial division [31]. Importantly, mitochondria undergoing fission events following primary lysosome-to-mitochondria contact remained functional, and did not undergo mitophagy [31]. In line with these published observations, we found that mitochondrial fission events in pMSCs of early 1st trimester produce small, but functional organelles, facilitated by primary lysosome-to-mitochondrial contact.

Various studies have highlighted the importance of lipid homeostasis in mediating mitochondrial dynamic events [42, 43]. We reported that alterations in the sphingolipidome are an important determinant for accelerated trophoblast cell death in PE [24]. Specifically, mitochondrial ceramide build-up [24] was responsible for tilting the balance towards fission in preeclamptic trophoblast cells [10]. In line with these findings, we observed that mitochondria isolated from preeclamptic pMSCs have also elevated ceramide levels, underscoring a role for ceramide in promoting mitochondrial fission.

Mounting evidence has recognized the contribution of exosomes in cell-to-cell communication during placentation [44], with the majority of studies focusing on trophoblast derived exosomes [45, 46]. The current study found that PE pMSC-derived exosomes increased mitochondrial fission in recipient trophoblastic JEG3 cells. This finding prompted investigation into the exosome cargo, which revealed increased amounts of sphingomyelin in PE pMSC-derived exosomes. Sphingomyelin enrichment of PE-pMSC exosomes is likely responsible for the increased mitochondrial fission in recipient JEG3 cells as JEG3 exposure to SM and/or overload of SM *via* treatment with SMPD1 inhibitors produced the same effect. Furthermore, we found that SM 18:0 increased p-DRP1 expression and distribution in JEG3 cells and that MDiVi-1 treatment of JEG3 cells reversed the sphingomyelin-induced increase in ER-to-mitochondria tethering, and thus also fission. To our knowledge, this is the first report of sphingomyelin influencing the expression and distribution of DRP1, a key factor in mitochondrial fission.

In conclusion, mitochondrial fission is augmented in pMSCs isolated from early 1^st^ trimester and preeclamptic placentae, which both experience hypoxia. In preeclamptic pMSCs, hypoxia-mediated fission is accompanied by exuberant ROS production and activation of the mitophagy machinery, ultimately contributing to the heightened cell death that typifies PE. In contrast, low-oxygen-mediated fission in early 1^st^ trimester pMSCs appears to be an oxygen-sparing, adaptive mechanism characterized by lower levels of ROS production, with no indication of significant mitophagy. Exosomes released by preeclamptic pMSCs negatively influence the mitochondrial homeostasis of neighboring trophoblast cells by stimulating fission, thereby promoting cell death. As hypoxia-dependent mitochondrial fission is mediated by DRP1, MDiVi-1 therapy could be considered to correct impaired mitochondrial dynamics and associated cell fate events in PE.

## Supplementary information

Supplementary Table 1

Supplementary Table 2

Supplementary Figures

Supplementary Methods

Original Western Blots

Reproducibility checklist

## Data Availability

Data included in the paper are available from the corresponding author upon request.
