# Supplementary material for: Dichotomy in hypoxia-induced mitochondrial fission in placental mesenchymal cells during development and preeclampsia: consequences for trophoblast mitochondrial homeostasis
Source: Cell Death Dis. 2022 Feb 26;13(2):191. doi: 10.1038/s41419-022-04641-y (PMC8882188; doi:10.1038/s41419-022-04641-y)
Supplement: Supplementary file 1 — Supplementary Table 1 [file 41419_2022_4641_MOESM1_ESM.docx]

**Supplementary Table 1** Clinical features of preeclamptic and normotensive controls

|  | **PE**  **(n=7)** | **Control**  **(n=9)** | **p-value** |
| --- | --- | --- | --- |
| Gestational Age at Delivery (weeks) | 30.33 ± 1.15 | 36.7 ± 3.70 | p<0.001 |
| Pre-term birth (%) | 100% | 33.33% |  |
| Fetal Birth Weight (grams) | 1084.29 ± 193.89 | 2973.33 ± 780.50 | p<0.001 |
| Fetal Sex | 28.57% F  71.43% M | 44.44% F  55.56% M | ns |
| Blood Pressure (mmHg, S/D) | S: 174.57 ± 30.40^C^  D: 112.14 ± 21.62^C^ | S: 118.33 ± 7.76  D:69.83 ± 9.24 | p<0.001 |
| Proteinuria (grams/day) | 1.57 ±1.61 | n/a |  |
| Mode of Delivery (%) | 100% C/S^A^ | 44.44% C/S  55.56% VS | ns |

Data are presented as mean ± SEM. A nonparametric Mann-Whitney test was used when comparing gestational age at delivery, fetal birth weight, and blood pressure in PE versus control, and a 𝜒^2^ test was used when comparing fetal sex and mode of delivery in PE vs control. Significance, p<0.05; ns, non-significant.

PE, preeclampsia; F, female; M, male; S, systolic; D, diastolic; CS, caesarian section; VS, vaginal delivery.
