# Supplementary material for: Dichotomy in hypoxia-induced mitochondrial fission in placental mesenchymal cells during development and preeclampsia: consequences for trophoblast mitochondrial homeostasis
Source: Cell Death Dis. 2022 Feb 26;13(2):191. doi: 10.1038/s41419-022-04641-y (PMC8882188; doi:10.1038/s41419-022-04641-y)
Supplement: Supplementary file 2 — Supplementary Table 2 [file 41419_2022_4641_MOESM2_ESM.docx]

**Supplementary Table 2** Measurement of mitochondrial parameters in first trimester primary isolated placental mesenchymal stromal cells.

|  | **6-8 week pMSC**  **(n=4)** | **10-12 week pMSC**  **(n=3)** | **p-value** |
| --- | --- | --- | --- |
| Number of images | 37 | 42 |  |
| Number of mitochondria | 415 | 287 |  |
| Number of mitochondria/image | 10.84 ± 1.270 | 6.783 ± 0.640 | p<0.05 |
| Surface area (µm^2^) | 0.100 ± 0.026 | 0.206 ± 0.032 | p<0.05 |
| Perimeter (µm) | 1.3243 ± 0.189 | 2.04 ± 0.209 | p<0.05 |
| Ferret’s diameter (µm) | 0.471 ± 0.078 | 0.824 ± 0.085 | p<0.05 |
| Aspect ratio | 0.652 ± 0.642 | 2.732 ± 0.101 | p< 0.05 |
| Circularity (0-1) | 0.769 ± 0.019 | 0.644 ± 0.017 | p<0.01 |

pMSC: placental mesenchymal stromal cells.

Data were obtained from TEM images and values are expressed as mean ± SEM . Data were analyzed using unpaired student’s t-test.
