# Supplementary material for: Dichotomy in hypoxia-induced mitochondrial fission in placental mesenchymal cells during development and preeclampsia: consequences for trophoblast mitochondrial homeostasis
Source: Cell Death Dis. 2022 Feb 26;13(2):191. doi: 10.1038/s41419-022-04641-y (PMC8882188; doi:10.1038/s41419-022-04641-y)
Supplement: Supplementary file 3 — Supplementary Figures [file 41419_2022_4641_MOESM3_ESM.pdf]

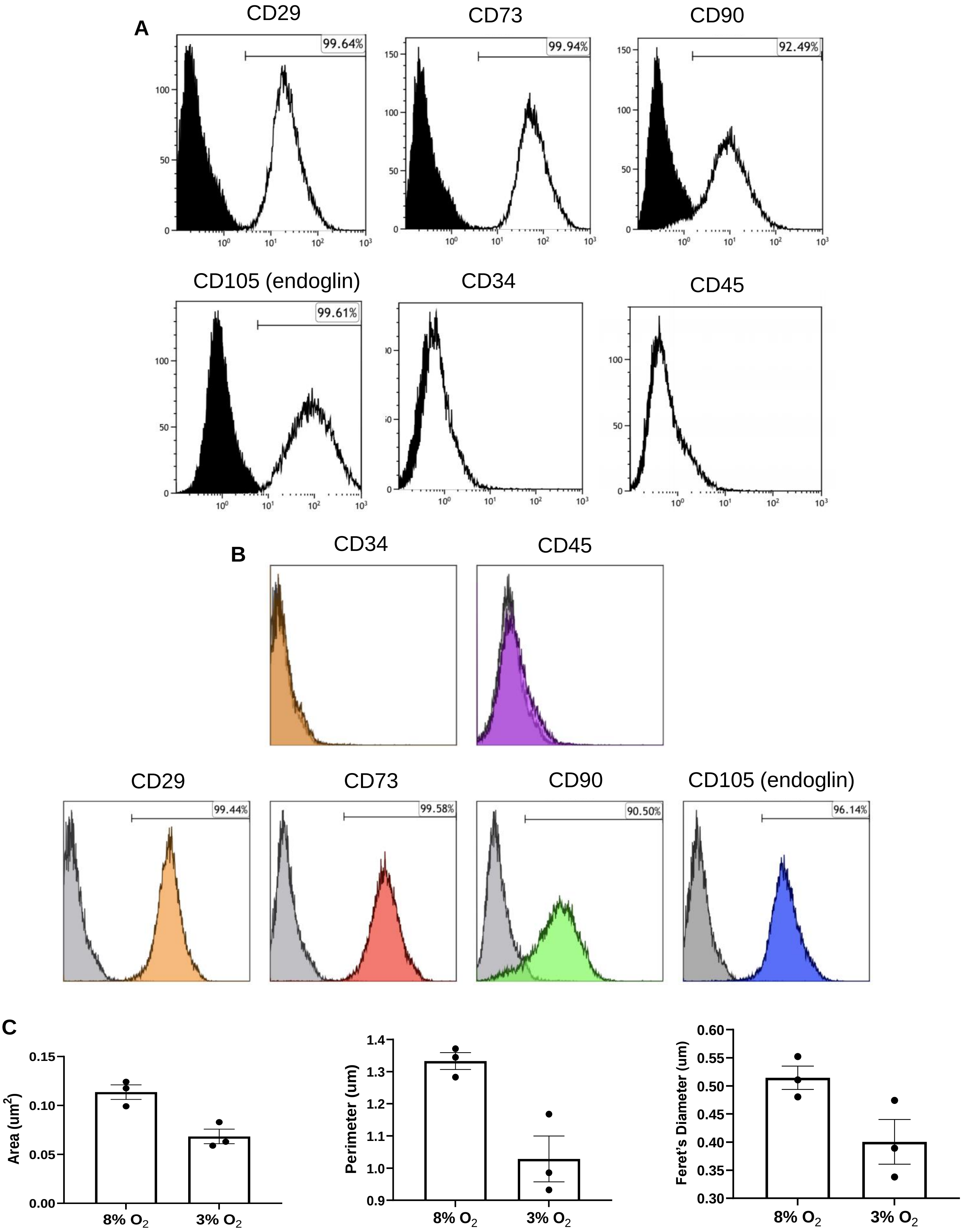


**Supplementary Figure 1. Characterization of human placental mesenchymal stromal cells.** Representative FACS profiles for first trimester (**A**) and PE (**B**) pMSCs are shown. The isolated pMSCs are positive for mesenchymal stromal markers CD29, CD73, CD90 and CD105 (Endoglin) and negative for hematopoietic markers CD34 and CD45. Every separate isolation of pMSCs was characterized by flow cytometry. (**C**) Quantification of mitochondrial morphology (surface area, perimeter and Feret's diameter) in term pMSCs cultured at 3% and 8% $O_2$ conditions.

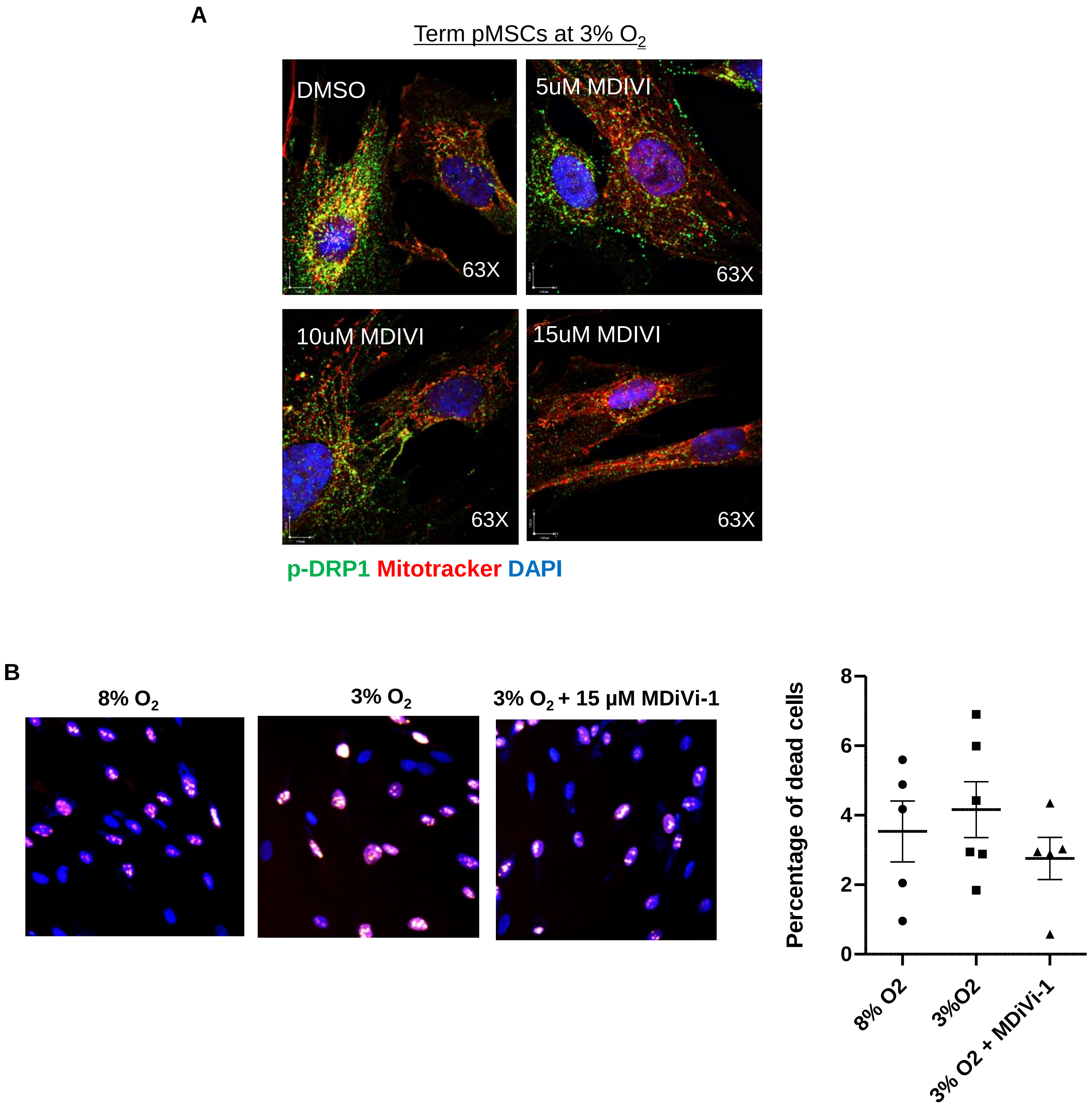


**Supplementary Figure 2. p-DRP1 localization in term pMSCs following treatment with various concentrations of MDiVi-1. (A)** Representative immunofluorescence confocal images of p-DRP1 (green) and Mitotracker (red) in pMSCs treated with either 5-15 uM MDiVi-1 or vehicle (DMSO) for 24-hours. Nuclei are stained with DAPI (blue). Term pMSCs treated with 15uM MDiVi-1 showed maximum decrease of pDRP1-co-localization with Mitotracker. (**B**) Representative images of Ki67 staining and associated densitometry in term pMSCs maintained at 8%$O_2$ and 3%$O_2$ with and without MDiVi-1 (15μM). MDiVi-1 treatment did not affect the proliferation of the cells.

**A**

**PTC** **PE**

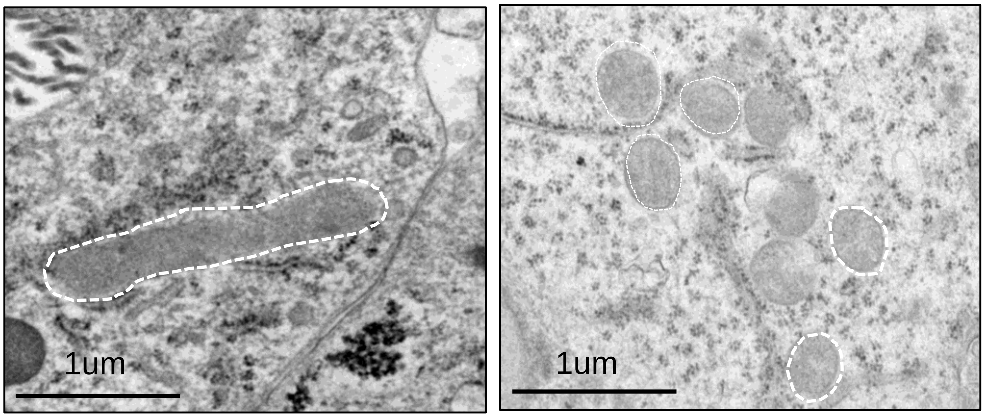


**B**

| | PTC (n=3) | PE (n=3) | p- value |
|---|---|---|---|
| Surface area ($\mu m^2$) | 2.942 ± 0.239 | 1.416 ± 0.036 | p<0.01 |
| Perimeter (µm) | 2.583 ± 0.268 | 1.47 ± 0.088 | p<0.05 |
| Feret's diameter (µm) | 1.035 ± 0.146 | 0.535 ± 0.040 | p<0.05 |
| Aspect ratio | 0.339 ± 0.045 | 0.150 ± 0.0165 | p< 0.05 |
| Circularity (0-1) | 0.642 ± 0.033 | 0.852 ± 0.010 | p<0.01 |

**Supplementary Figure 3. Mitochondrial morphology of villous mesenchymal cells in preeclamptic (PE) and normotensive control (PTC) placentae**. (**A**) Representative transmission electron microscopy (TEM) of placental mesenchymal cells within whole placental tissue sections from PTC (n=4) and PE (n=4) pregnancies. Scale bar indicates size. (**B**) Measurement of mitochondrial parameters (surface area, perimeter, Ferret's diameter, aspect ratio, and circularity) in mesenchymal cells within whole placental tissue sections from PE and PTC placentae. Data were obtained from TEM images (n=3 different placentae per condition; 4 sections/placentae; 4 images/section). Values are presented as mean ± SEM. Significance was determined using unpaired Student's T-test.

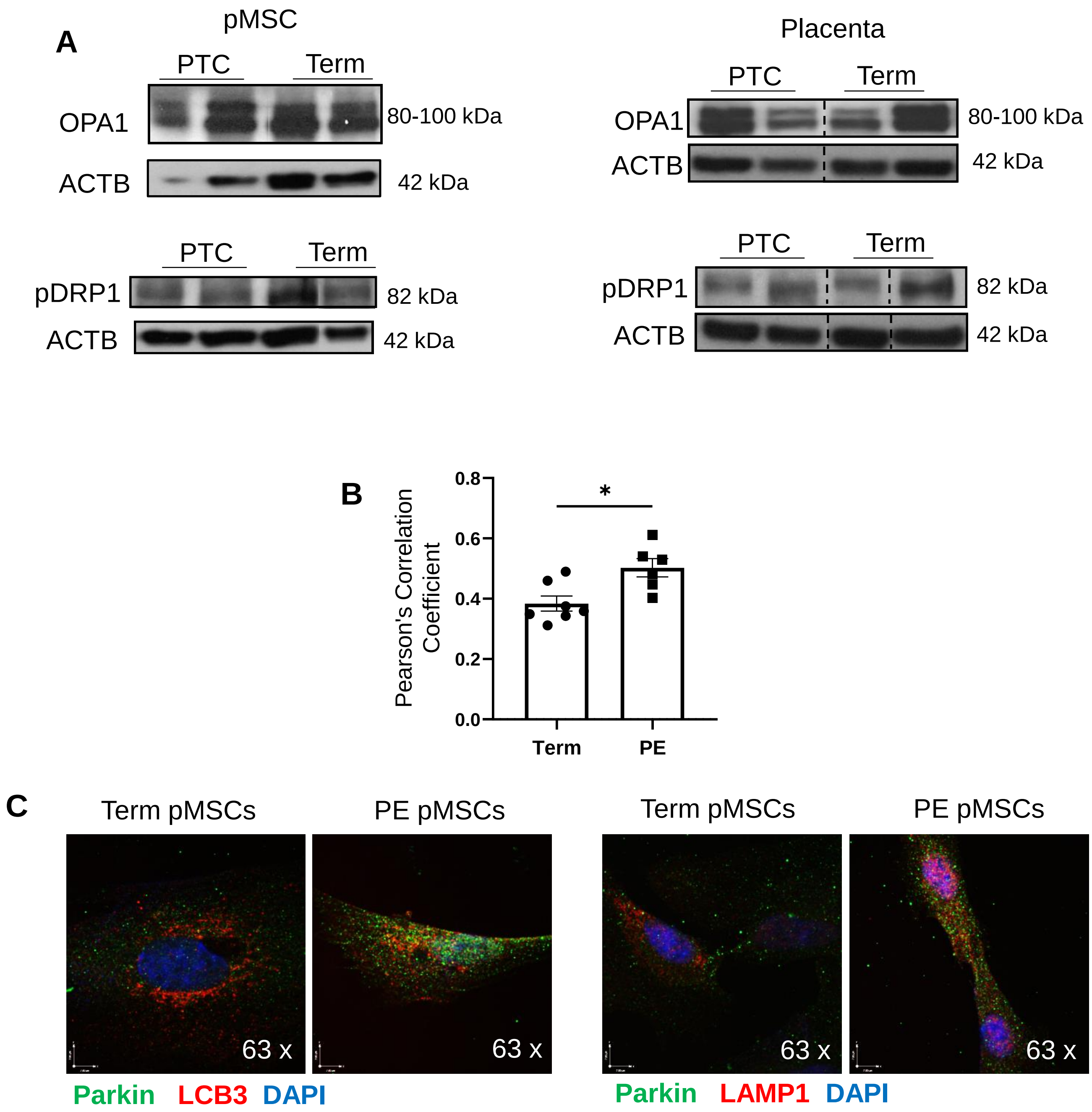


**Supplementary Figure 4. Mitophagy adaptor proteins are overexpressed in the mitochondrial fraction in PE pMSCs.** (**A**) Representative WB for OPA1 nad pDRP1 and control ACTB in pMSC (left panel) and whole placenta lysate (right panel) from term and preterm controls (PTC). **(B)** Pearson's Correlation Coefficient analysis of PINK1 co-localization with Mitotracker in term and PE pMSCs **(C)** Representative immunofluorescence confocal images of Parkin (green) and LC3B (left panel, red) or LAMP1 (right panel, red) in term and PE pMSCs. Nuclei are stained with DAPI (blue).

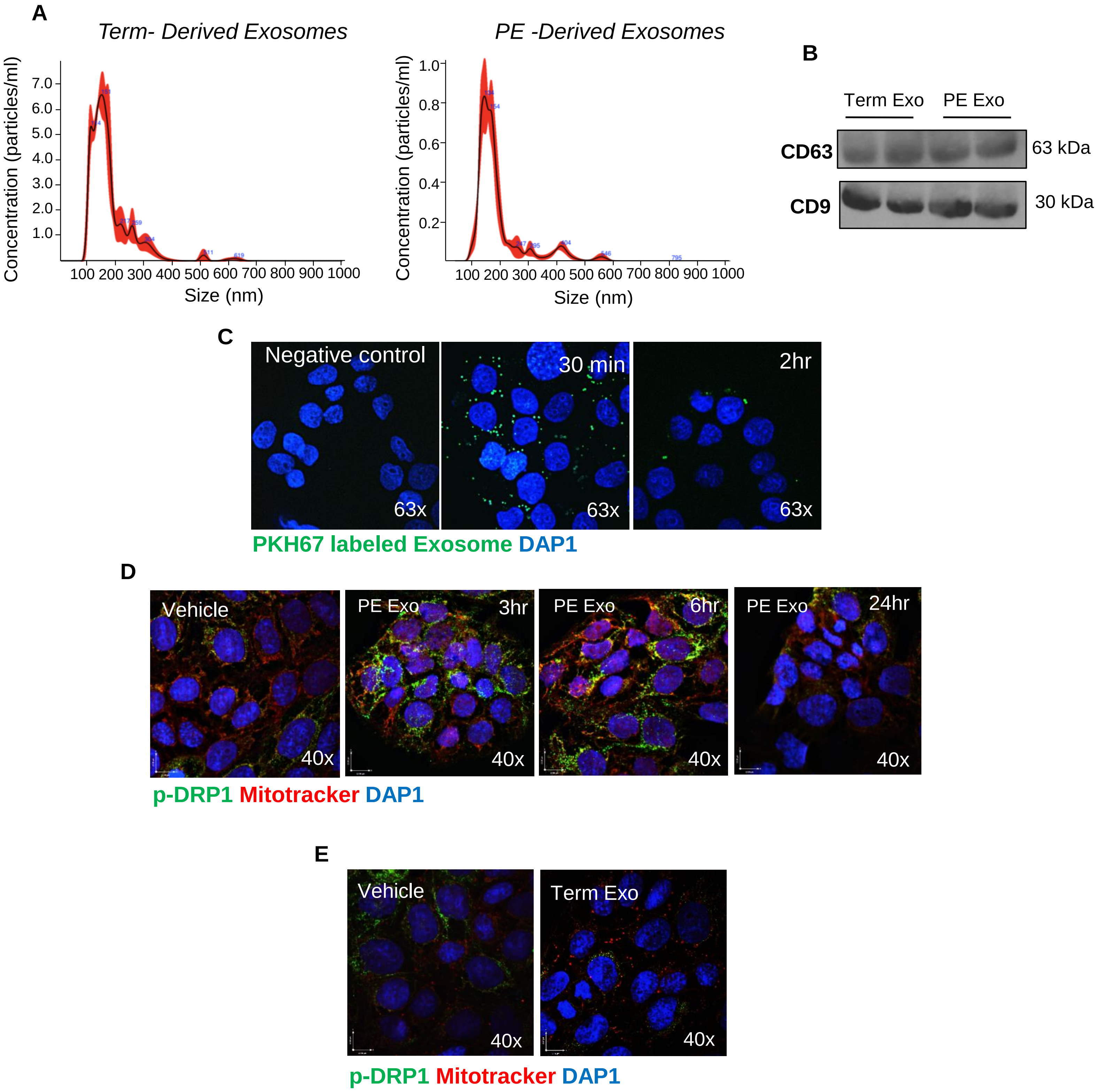


**Supplementary Figure 5. Exosome characterization and effect on mitochondrial dynamics in recipient JEG3 cells.** (**A**) Representative nanosight particle tracking analysis of purified exosomes secreted by term and PE pMSC (n=4 separate isolations). (**B**) Representative Western blots for exosomal markers CD63 and CD9 in purified exosomes isolated from term and PE pMSCs (n=4 separate isolations). **(C)** Representative immunofluorescence confocal images of JEG3 cells exposed to PKH67 (green)-labelled purified exosomes secreted by term PMSCs for 30 and 120 minutes. Nuclei are stained with DAPI (blue). Unlabelled exosomes are used as negative control. Experiments were repeated with similar results twice. (**D**) Representative immunofluorescence confocal images of p-DRP1 (green) and MitoTracker (red) in JEG3 cells treated with either vehicle (PBS, 24 hours) or exosomes from PE pMSCs (PE Exo). JEG3 cells are treated for 3, 6, and 24 hours to determine the optimal effect on p-DRP1 co-localization to the mitochondria. (**E**) Immunofluorescence confocal images of p-DRP1 (green) and Mitotracker (red) in JEG3 cells treated with either Vehicle (PBS, 24 hours) or exosomes from term pMSCs. Nuclei are stained with DAPI (blue).

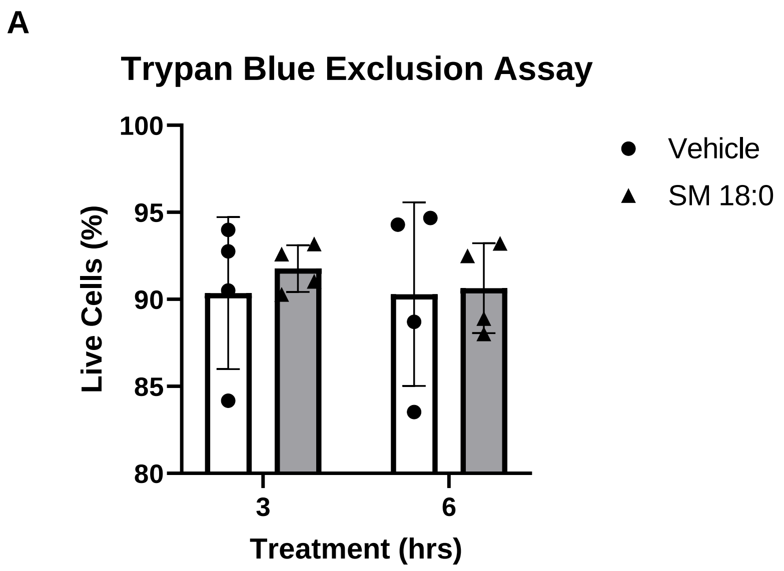


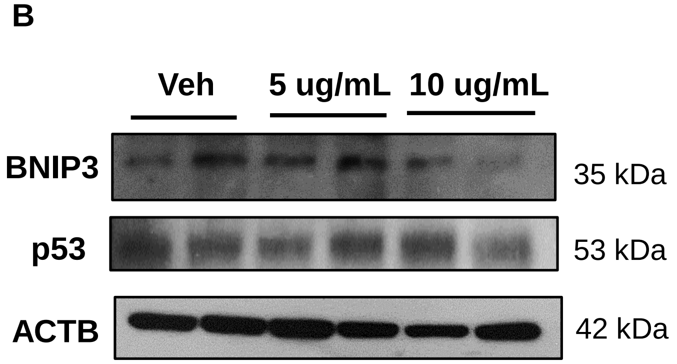


**Supplementary Figure 6. Sphingomyelin treatment does not affect cell death in JEG3 cells. (A)** Trypan blue exclusion assay (displayed as percentage of live cells) in JEG3 cells exposed to 10 ug/mL sphingomyelin for 3 or 6 hours. **(B)** WB for BNIP3 and p53 in JEG3 cells following 10 ug/mL sphingomyelin treatment for 6 hours.
