# Supplementary material for: Dichotomy in hypoxia-induced mitochondrial fission in placental mesenchymal cells during development and preeclampsia: consequences for trophoblast mitochondrial homeostasis
Source: Cell Death Dis. 2022 Feb 26;13(2):191. doi: 10.1038/s41419-022-04641-y (PMC8882188; doi:10.1038/s41419-022-04641-y)
Supplement: Supplementary file 4 — Supplementary Methods [file 41419_2022_4641_MOESM4_ESM.pdf]

**Supplementary Materials and Methods**

***Placental Mesenchymal Isolation and Culture***

Placental tissue was subjected to a series of washes with Hanks's balanced salt solution (HBSS without $Ca^{2+}$ and $Mg^{2+}$) followed by enzymatic digestions: 30 min in digestion buffer 1 (200mL Dulbecco's Modified Eagle Medium (DMEM), 0.25 g Trypsin, 0.03-0.04g DNase) and 2 x 30 min in digestion buffer 2 (350mL DMEM and 0.45 g trypsin), and final 2 x 20-min collagenase digestions. The supernatant, containing pMSCs, was neutralized with DMEM + 10% (v/v) fetal bovine serum (FBS), centrifuged at 240*g* for 5 min, and the cell pellet was resuspended in wash buffer (DMEM + 2% (v/v) FBS). Cells were filtered through a 70 μm filter, layered on top of a Percoll gradient (5-70% Percoll diluted in PBS) and centrifuged at 425*g* for 10 min. Cells that settled in the middle of the gradient (~10mL) were collected and pelleted by centrifugation at 3,500*g* for 10 min. Cells were resuspended in 10mL culture media (DMEM plus 10% (v/v) FBS and 1% (v/v) penicillin/streptomycin (P/S)), plated and allowed to grow in $75cm^2$ tissue culture flasks at 37°C. Cells (pMSCs) were cultured at an oxygen tension that corresponded to their physiological oxygen environment at time of isolation. Term or PE pMSCs cultured at 8% and 3% $O_2$ were treated with either DMSO vehicle or Mitochondrial Division Inhibitor 1 (MDiVi-1; M0199-5MG, Millipore Sigma, St. Louis, MO, USA), a selective cell permeable inhibitor of DRP1 oligomerization and GTP activity[1] at a concentration of 15μM.

***pMSC-derived Exosome Isolation and characterization***

For pMSCs exosome isolation, conditioned media was collected and centrifuged at 12,000*g* for 45 min to pellet larger vesicles. The supernatant was then spun for 16 hours at 100,000*g*. The exosome pellet was suspended in PBS and filtered through a 0.22 μM filter. Effectiveness of each exosome

preparation was determined by Western blotting (WB) for exosome markers CD63 and CD9[2]. Additionally, Nanosight Tracking Analysis (NTA) was performed to confirm exosome size and concentration.

***Exosome uptake by JEG3 cells***

The PKH67 Green Fluorescent Cell Linker Kit (Sigma, PKH67GL-1KT) was used to stain exosomes. Isolated exosomes were incubated in dye solution in the dark as per manufacturer's instructions. An equal volume of 1% (w/v) bovine serum albumin was added to stop the staining reaction and to bind excess dye. Labeled exosomes were pelleted (120,000*g* for 60 min) and resuspended in PBS. The spin and wash cycles were repeated 3 times. The fluorescent-labeled exosomes were then added to JEG3 cells and exosomal uptake was assessed at 30-120 min intervals using a Leica SD6000 spinning disk confocal microscope (Leica Camera, Wetzlar, Germany).

***Fluorescent Activated Cell Sorting (FACS)***

pMSCs were harvested and strained using a 40 μm cell strainer. Single cells were resuspended in staining buffer ((Hank's balanced salt solution supplemented with 2% (v/v) FBS and 10 mM HEPES) and subsequently incubated for 30 min with antibodies validated for flow cytometry at a 1:100 dilution. Anti-human CD29-PerCP-ef710, CD34-PerCP-ef710, CD45-ef450, CD73-PE, CD90-FITC and CD105-APC were all purchased from eBiosciences-ThermoFisher Scientific®. Stained cells were washed with staining buffer and centrifuged at 1000*g* for 10 min. Stained cells were then resuspended in 400 μl of staining buffer and analyzed using the Beckman Coulter Galios flow cytometer with data analysis completed using Kaluza software (Beckman Coulter,

Mississauga, ON, Canada). Unstained cells were used as negative controls for proper gating and voltage setting as per manufacturer's recommendations.

***Transmission Electron Microscopy (TEM)***

Placental tissue from PE and normotensive age-matched control pregnancies were fixed in 2.5 % (v/v) glutaraldehyde in 0.1 M cacodylate buffer (pH 7.3). In case of pMSCs, cells were first pelleted at 300*g* for 5 min at 4°C and then fixed in 2.5% (v/v) glutaraldehyde. Tissue and pMSC samples were then processed for TEM analysis at the Nanoscale Biomedical Imaging Facility at The Hospital for Sick Children, Toronto as reported[3]. Images were obtained using a FEI Tecnai 20 transmission electron microscope (FEI Life Sciences-Thermo Fisher Scientific®). TEM micrographs of pMSCs from 1$^{st}$ trimester (n=7), term (n=4) and PE (n=4) placentae were analyzed for mitochondrial shape and size using ImageJ software by manually tracing only clearly discernable outlines of mitochondria as previously described[3]. For each placenta or pMSC isolation, 4 representative images were obtained, and mitochondria morphology was assessed. Lysosomes were identified based on previously well-established features: a well-defined singular, homogenous lipid-bilayer membrane that is electron dense relative to the surrounding cytosol[4, 5]. Additionally, primary and secondary lysosomes were distinguished based on the relative electron density and granularity of the luminal matrix. Primary lysosomes were more electron dense and granular while secondary lysosomes were electron lucent and contained less granules[5].

***Immunofluorescence***

Following respective treatments, pMSCs were fixed as previously described [3]. Primary antibodies diluted in antibody diluent (0.4% (w/v) sodium azide, 0.625% (w/v) gelatin) and 5% (v/v) normal

horse serum) were added to fixed cells for overnight incubation in 4°C. Cells were then washed 3 times in PBS and incubated for 1 hour with Alexa Flour®-conjugated secondary antibody appropriate for respective species of primary antibodies (Invitrogen, Carlsbad, CA, USA). Nuclei were visualized with 4',6-diamino -2-phenylindole (DAPI). Coverslips were then mounted with Immuno-Mount™ (ThermoFisher Scientific®, Waltham, Massachusetts) and imaged using a Leica SD6000 spinning disk confocal microscope (Leica Camera, Wetzlar, Germany).

***Antibodies***

Primary antibodies against DRP1 (H-300, sc-32898, rabbit anti-human [WB 1:1000], TOM20 (FL - 135, sc - 11415, rabbit anti-human [WB 1:1500]), ACTB (C-4 sc-47778, mouse anti-human [WB 1:2000], CD63 (E-12, sc 365604, mouse anti-human [WB 1:500] and CD9 (C-4, sc13118, mouse anti-human [WB 1:500] were purchased from Santa Cruz Biotechnology, Dallas, Texas. p-DRP1 (S616) (3455S, rabbit anti-human [IF: 1:500, WB 1:1000]) antibody was purchased from Cell Signalling Technology®, Danvers, Massachusetts. OPA1 (612607, mouse anti-human [WB 1:1000]) was purchased from BD Biosciences®, San Jose, California. PINK1 (BC100 - 494, rabbit anti-human [WB 1:1200]) was purchased from Novus Biologicals®, Littleton, Colorado. Parkin (AB9244, rabbit anti-human [IF 1:500]) was purchased from Millipore Sigma®, Darmstadt, Germany. Mouse anti-human VDAC1 (ab14734) [WB 1:1000] was purchased from Abcam, Cambridge, UK. Horseradish peroxidase (HRP) conjugated rabbit anti-mouse (1:2000) and HRP goat anti-rabbit (1:000) secondary antibodies used for WB were from Santa Cruz Biotechnology. Alexa Fluor® 488 donkey anti-rabbit (A21206) and Alexa Fluor® 488 donkey anti-mouse (A-21202) secondary antibodies used for IF were also from Santa Cruz Biotechnology.

***Proximity Ligation Assay***

For negative controls, either one of the primary antibodies VDAC1 (ab14734, Abcam, Cambridge, UK), IP3R (ab5804, Abcam, Cambridge, UK). RAB7 or TOM20 (FL-135, sc-11415, Santa Cruz Biotechnology) or one of the plus-PLA or minus-PLA probes was omitted. An interaction is indicated by the appearance of a red fluorescent dot.  IP3R and VDAC interactions were quantified using ImageJ software (https://imagej.nih.gov/ij/). Briefly, following separation of each image into the red (TRITC) and blue (DAPI) fluorescent channels, the number of individual red puncta per field was measured using the 'Analyze particles' command within a specified region of interest. Quantification was performed for a minimum of three images obtained from several independent experiments (i.e. six term control and three preeclamptic placentae), and represented as an average number of PLA puncta per cell.

***SMPD1 Activity Assay***

Thirty of total protein was prepared from JEG3 whole cell lysates. Samples and standards were loaded onto 96-well plates and incubated with the provided SMPD1 substrate at 37°C. Following incubation, stop buffer was added and samples were incubated for 10 minutes at room temperature with shaking. Plates were analyzed using a fluorescence microtiter plate reader (Tecan Infinite M200, Tecan US Inc., Morrisville, NC) at 360 nm excitation and 460 nm emission. A best-fit curve was generated using SMPD1 standards, and relative SMPD1 activity was determined by interpolation of slopes against the standard curve. Graphpad Prism software was employed for statistical analysis.

## *References*


1. Ruiz A, Alberdi E, Matute C. Mitochondrial Division Inhibitor 1 (mdivi-1) Protects Neurons against Excitotoxicity through the Modulation of Mitochondrial Function and Intracellular Ca(2+) Signaling. *Front Mol Neurosci* 2018, **11:** 3.

2. Ermini L, Ausman J, Melland-Smith M, Yeganeh B, Rolfo A, Litvack ML*, et al.* A Single Sphingomyelin Species Promotes Exosomal Release of Endoglin into the Maternal Circulation in Preeclampsia. *Sci Rep* 2017, **7**(1)**:** 12172.

3. Abbade J, Klemetti MM, Farrell A, Ermini L, Gillmore T, Sallais J*, et al.* Increased placental mitochondrial fusion in gestational diabetes mellitus: an adaptive mechanism to optimize feto-placental metabolic homeostasis? *BMJ Open Diabetes Res Care* 2020, **8**(1).

4. Aston D, Capel RA, Ford KL, Christian HC, Mirams GR, Rog-Zielinska EA*, et al.* High resolution structural evidence suggests the Sarcoplasmic Reticulum forms microdomains with Acidic Stores (lysosomes) in the heart. *Sci Rep* 2017, **7:** 40620.

5. Ermini L, Farrell A, Alahari S, Ausman J, Park C, Sallais J*, et al.* Ceramide-Induced Lysosomal Biogenesis and Exocytosis in Early-Onset Preeclampsia Promotes Exosomal Release of SMPD1 Causing Endothelial Dysfunction. *Front Cell Dev Biol* 2021, **9:** 652651.
