# Supplementary figures and images for: Dichotomy in hypoxia-induced mitochondrial fission in placental mesenchymal cells during development and preeclampsia: consequences for trophoblast mitochondrial homeostasis
Source: Cell Death Dis. 2022 Feb 26;13(2):191. doi: 10.1038/s41419-022-04641-y (PMC8882188; doi:10.1038/s41419-022-04641-y)

# Uncropped Blots

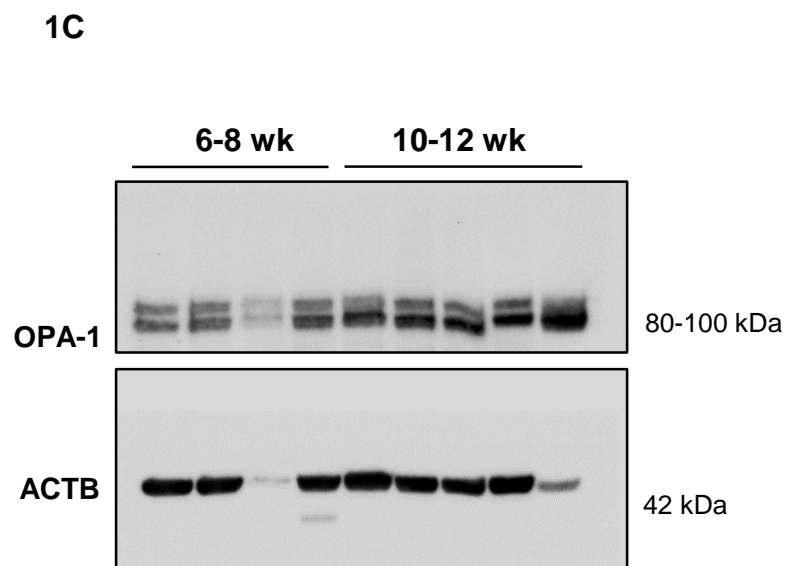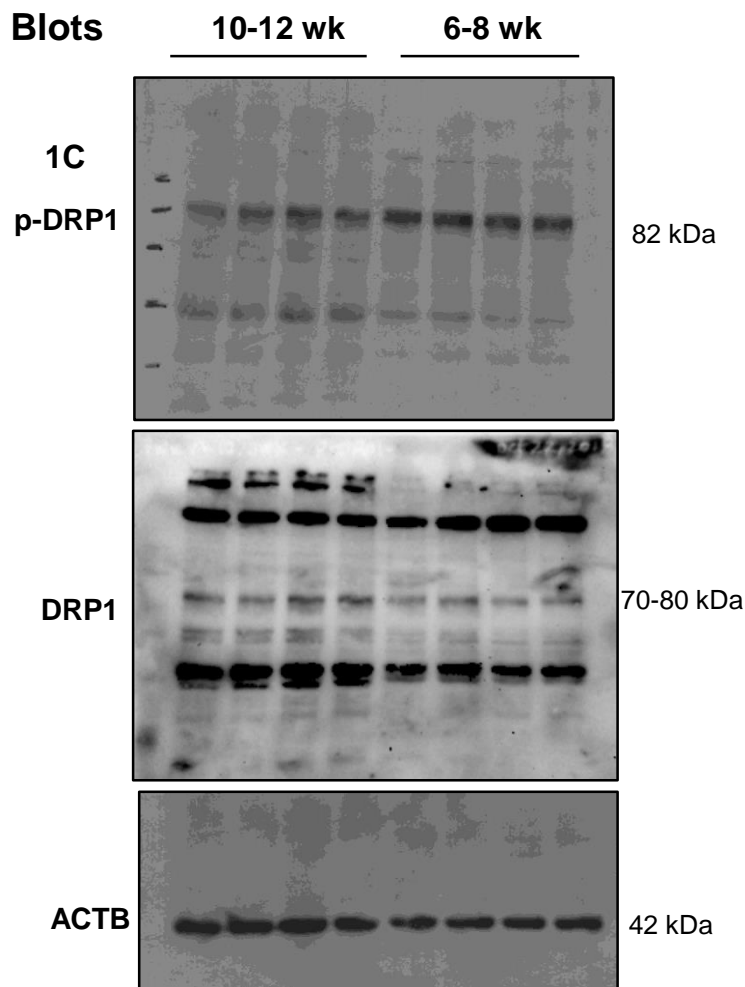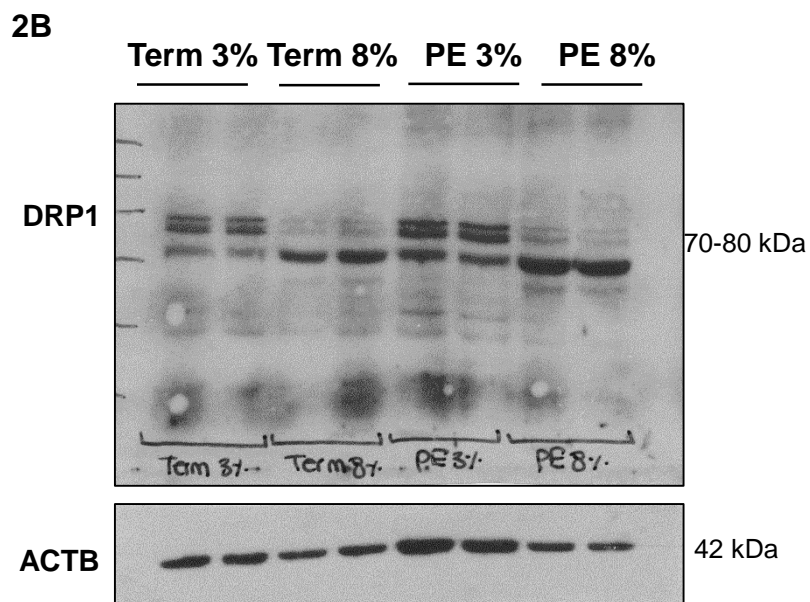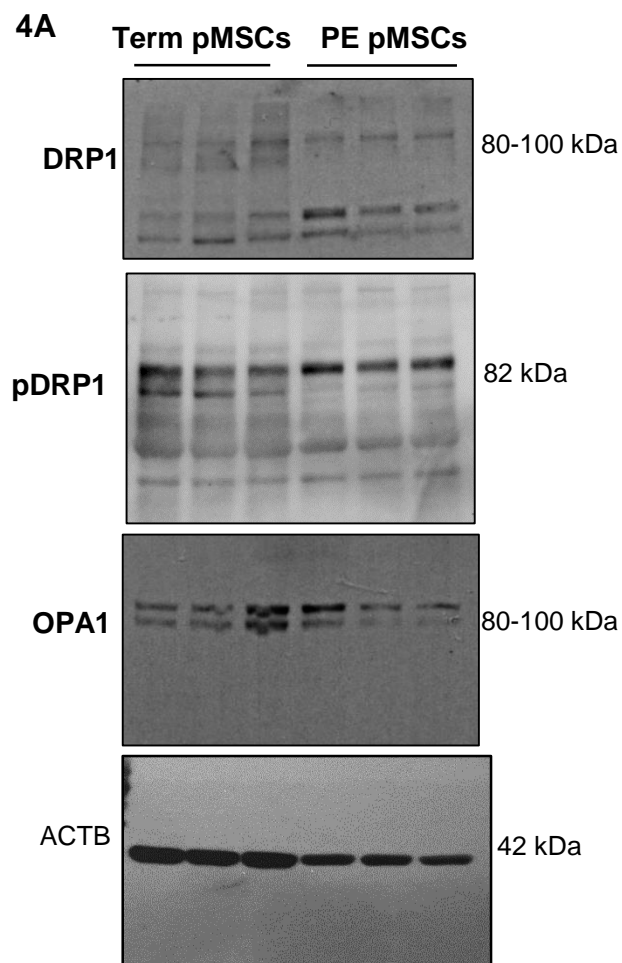

4B

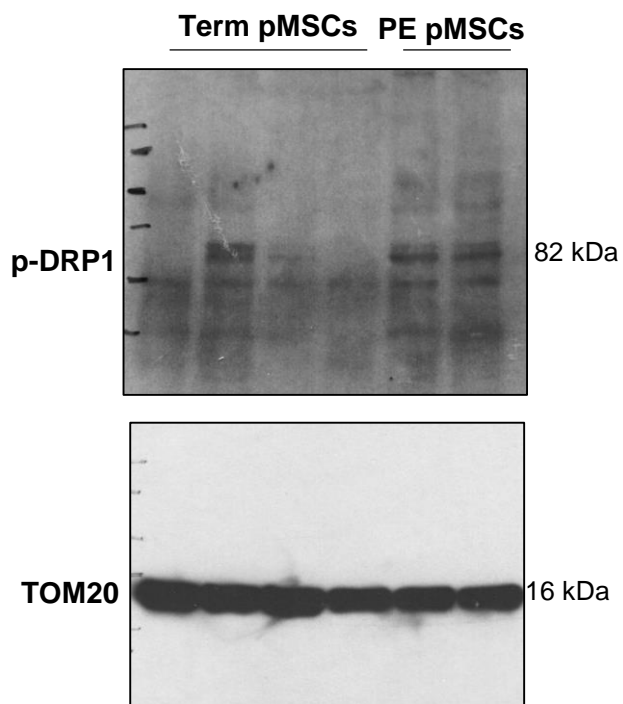

5C

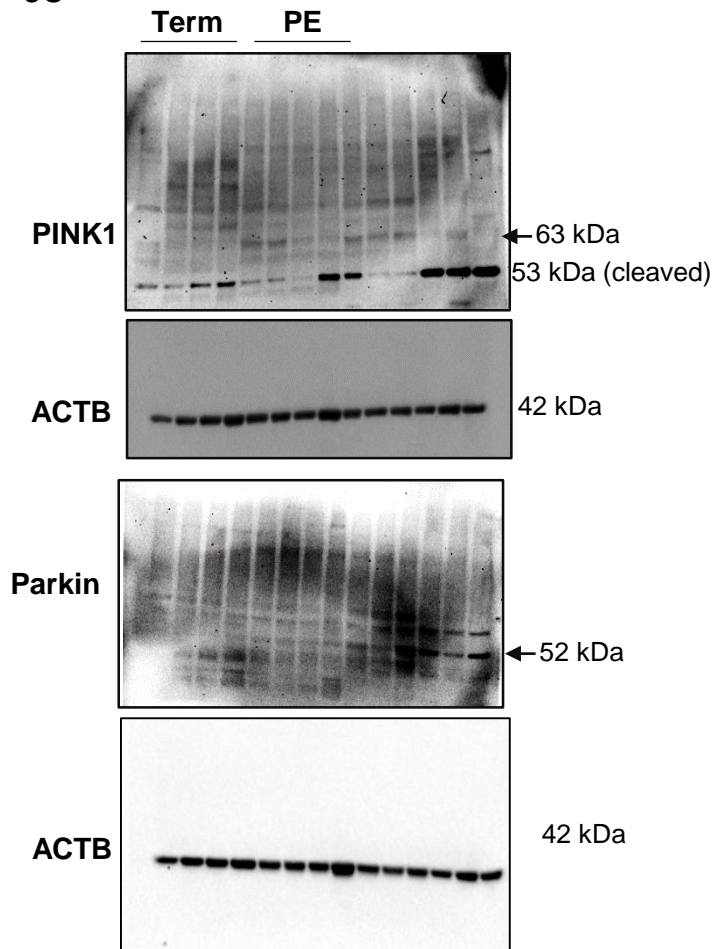

5E

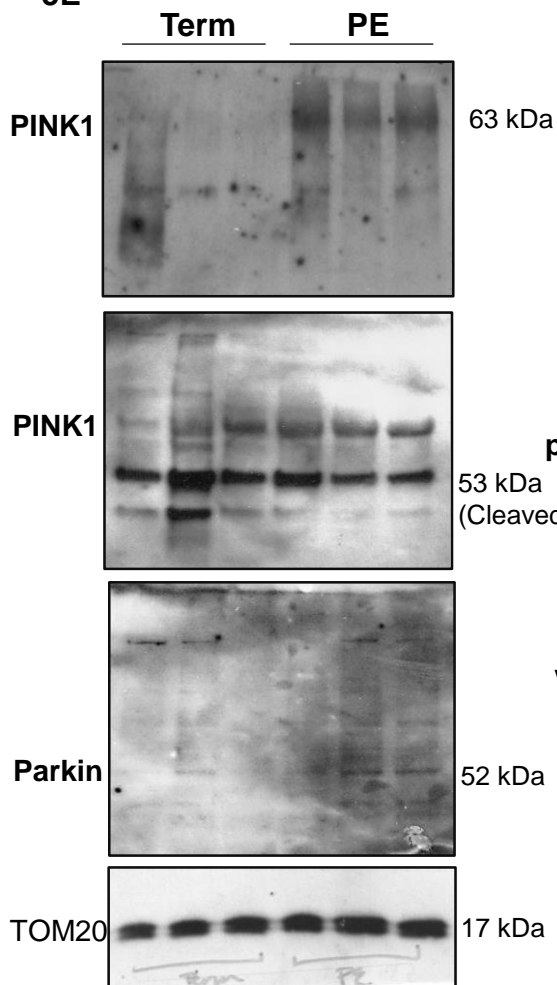

7B

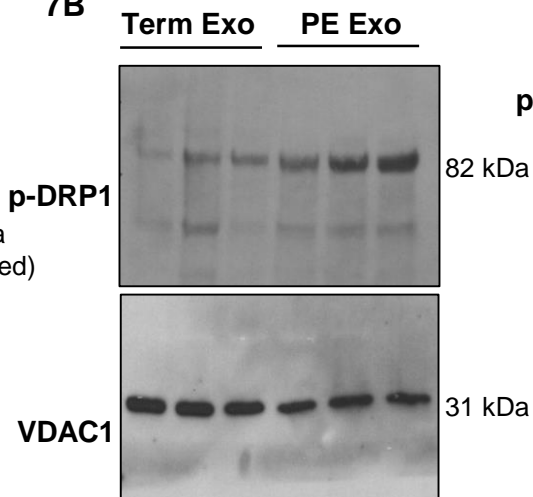

8B

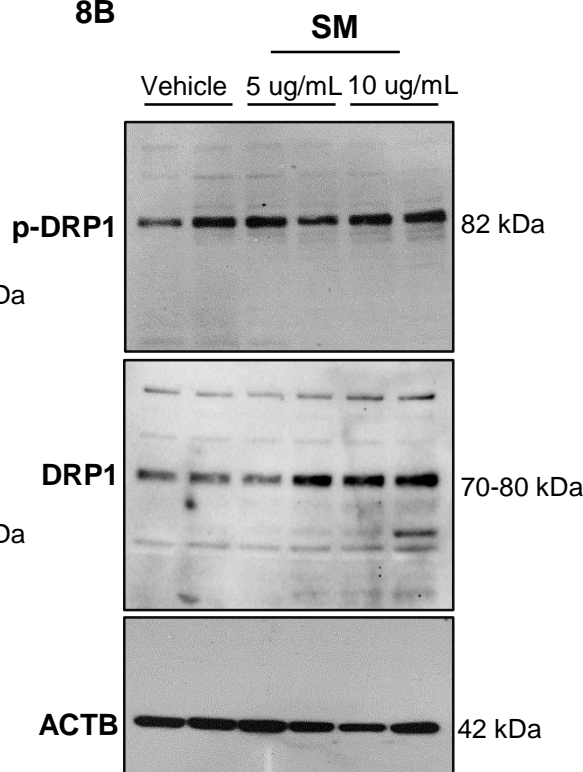

Supplementary Figure 4

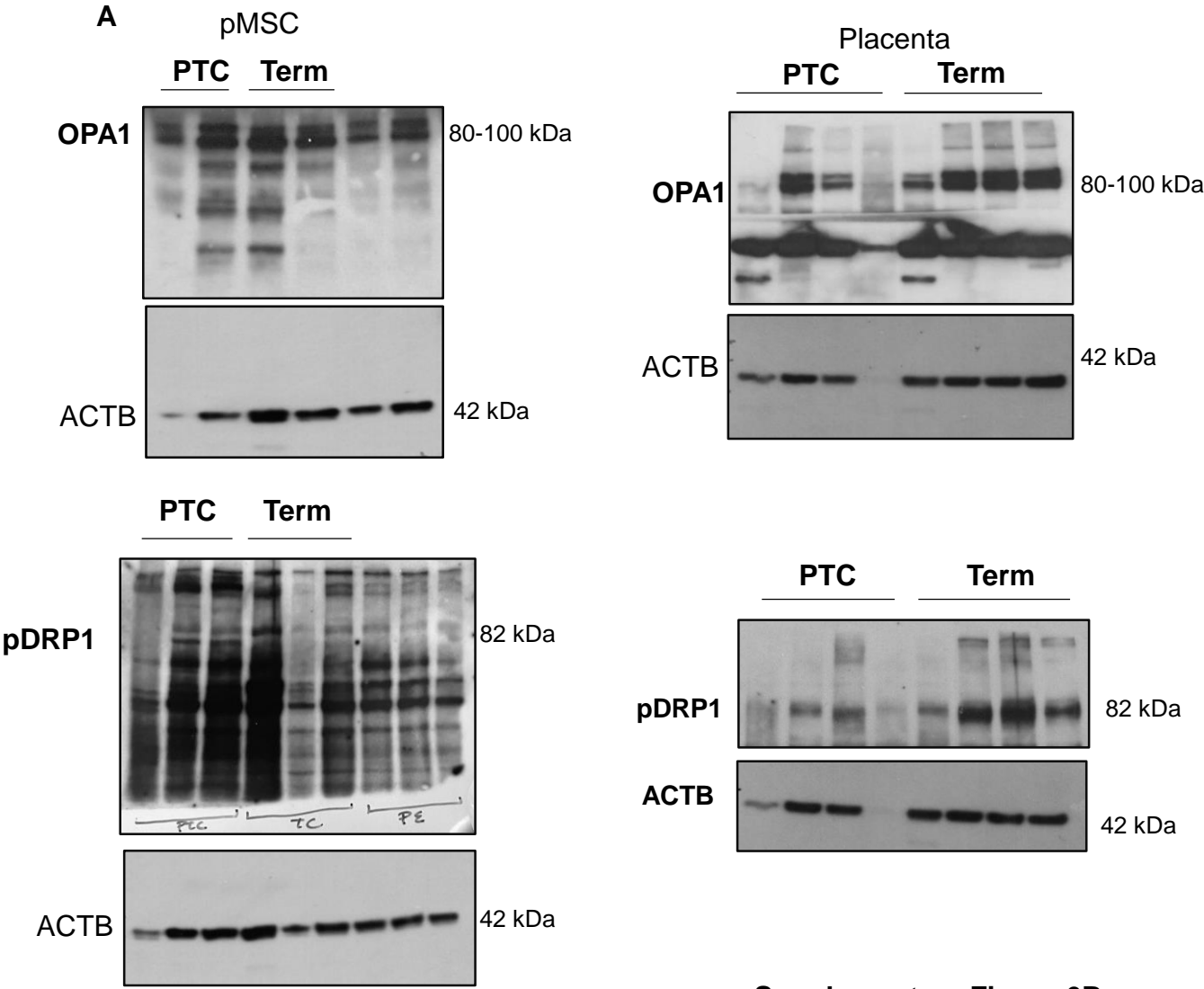

Supplementary Figure 6B

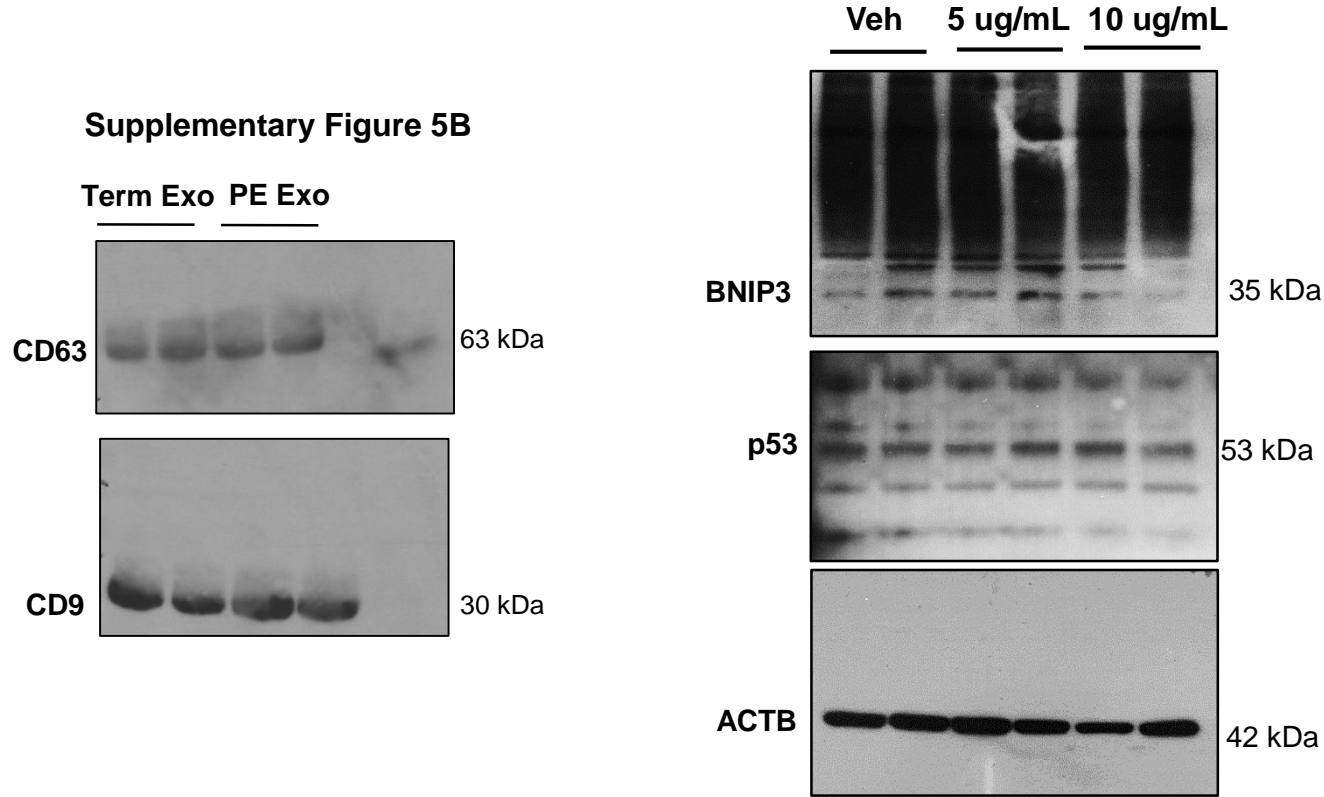

Supplement: Supplementary file 5 — Original Western Blots [file 41419_2022_4641_MOESM5_ESM.pdf]
